# Postpolymerization Modification by Nucleophilic Addition to Styrenic Carbodiimides

**DOI:** 10.1021/acsmacrolett.3c00382

**Published:** 2023-07-24

**Authors:** Hayden E. Houck, Kate A. McConnell, Conner J. Klingler, Adelle L. Koenig, Grace K. Himka, Michael B. Larsen

**Affiliations:** Department of Chemistry, Western Washington University, Bellingham, Washington 98225, United States

## Abstract

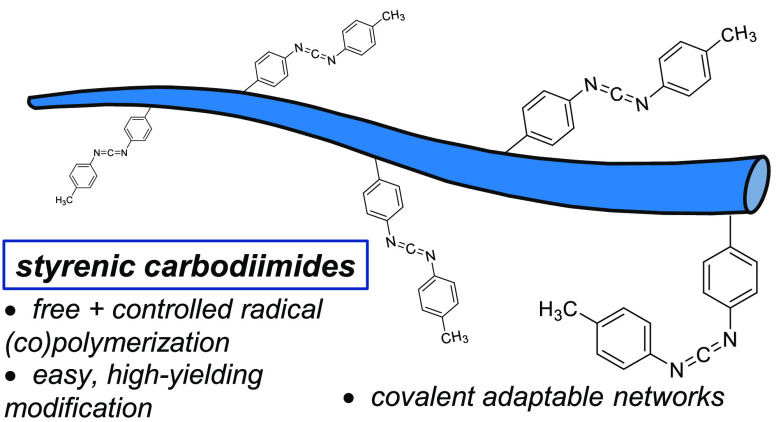

Carbodiimides are electrophilic functional groups that react with select nucleophiles under mild conditions. However, their potential as platforms for postpolymerization modification has been relatively underexplored. We describe the synthesis and radical polymerization of a styrenic carbodiimide which undergoes rapid nucleophilic addition with primary and secondary alkyl amines under ambient conditions, even in the presence of other protic nucleophiles. The monomer is amenable to both free and controlled radical (co)polymerization, and we further demonstrate the utility of this approach by preparing covalent adaptable networks through guanylation of the styrenic carbodiimide with difunctional amines. These materials exhibit a variation in relaxation times according to both the guanidine structure and concentration, providing a facile means for tuning dynamic behavior.

Together with controlled polymerization techniques possessing broad functional group tolerance, the synthetic modification of existing polymers enables the preparation of macromolecules with diverse functionalities and architectures.^1^ These complementary approaches each have their strengths: while it is often synthetically straightforward to polymerize a monomer containing a desired functional group, postpolymerization modification (PPM) can be the only method to install a given functionality if it is incompatible with the chosen polymerization conditions.^2^ Additionally, PPM facilitates diversification of a single polymer into a library of materials with varied properties^3−5^ and can result in self-assembly,^6,7^ cross-linking,^8^ or upcycling of commodity plastics.^9,10^

The unique synthetic and practical challenges associated with PPM, such as polymer isolation and removal of side products or byproducts, accentuate the need for modification reactions that occur with high fidelity and have straightforward purification procedures. Click chemistry is an immensely useful tool for PPM,^11^ but it can present complications such as unwanted catalyst complexation^12^ or require an excess of one reaction partner.^13,14^ Nucleophilic addition–elimination reactions of activated carbonyls are versatile PPM strategies,^15^ but byproducts formed from the leaving group may require removal by dialysis or multiple precipitations.^16−18^ In contrast, nucleophilic addition to polarized π bonds does not intrinsically require a leaving group, and the suitable choice of reaction partners can result in near-quantitative conversion without an added catalyst. For instance, the addition of amines to isocyanates occurs rapidly under ambient conditions, but the high reactivity of the isocyanate functional group toward other protic nucleophiles, such as adventitious water, limits its practical use as a PPM platform.^2^

Like isocyanates, carbodiimides (CDIs) are electrophilic heterocumulenes that undergo nucleophilic addition reactions.^19,20^ They are widely used in this context as dehydrating reagents in amide synthesis, and polymeric CDIs derived from step-growth polycondensation of diisocyanates^21^ are used as stabilizing additives for commercial polyesters and polyurethanes.^22^ Additionally, recent work demonstrated the preparation and modification of a new class of polymeric CDIs by Ir-catalyzed ring-opening metathesis polymerization.^23^ Aryl-substituted CDIs in particular occupy a useful niche of reactivity:^24^ they are bench-stable over the span of years, but react rapidly and quantitatively with primary or secondary alkyl amines under ambient conditions.^25^ While they have been explored as substrates for SmI_2_-mediated reductive coupling reactions,^26^ we envisioned styrenic aryl CDIs as a broadly useful PPM platform amenable to preparation by traditional radical polymerization methods. Here we present the free and controlled radical (co)polymerization of styrenic CDIs and their modification with alkyl amines. We show that the modification reaction can proceed to >95% conversion without added catalysts, such that the resulting polymer can be isolated simply through solvent removal. Additionally, we demonstrate the preparation of covalent adaptable network (CAN) materials by modifying copolymers with difunctional amines.

We first developed a synthetic route to styrenic CDI-containing monomer **1**, which required two steps from commercial starting materials (Schemes 1 and S1). Nucleophilic addition of 4-aminostyrene to 4-methylphenyl isothiocyanate followed by dehydrosulfurization of the intermediate thiourea using Mukaiyama’s reagent^27^ afforded **1** in multigram scales, with typical isolated yields of ∼70% over two steps. Subsequent free-radical polymerization yielded poly**1**, and all characterizations were consistent with the successful polymerization and retention of the CDI functionality (Figures S1–S5). SEC chromatograms indicated a polymer with a broad molar mass distribution (*M*_w_ = 129 kDa, *Đ* = 3.5), and ^1^H NMR spectroscopy was consistent with the proposed repeat unit structure. The FT-IR spectrum of poly**1** exhibited a significant absorption at ∼2100 cm^–1^, indicating a CDI stretching frequency. MALDI mass spectrometry of a lower molar mass sample showed the expected repeat unit spacing of 234 g/mol, with the primary populations possessing end groups derived from AIBN. Thermal characterization of the homopolymer indicated modest stability, with *T*_d,5%_ = 209 °C by TGA, and no *T*_g_ or other thermal transitions were detected by DSC prior to the onset of degradation.

**Scheme 1:**
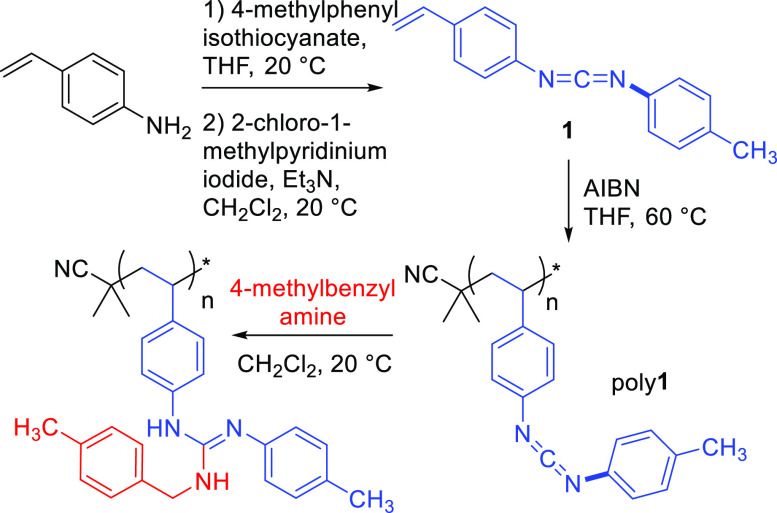
Synthesis and Modification of a Styrenic Carbodiimide Homopolymer

PPM was accomplished by combining poly**1** with an equimolar (vs repeat unit) amount of 4-methylbenzyl amine in CH_2_Cl_2_; no precautions were taken to exclude air or moisture during the reaction. After 1 h, FT-IR spectroscopy showed a complete disappearance of the CDI stretching frequency and the formation of absorbances at ∼1630 cm^–1^ attributed to guanidine C=N bonds (Figure S6). The resulting polymer was obtained in 94% yield by evaporation of volatiles, and characterization supported full conversion to the guanidine-containing repeat unit (Figures S7 and S8). The ^1^H NMR spectrum displayed resonances consistent with the new structure, and MALDI mass spectrometry indicated a change in repeat unit spacing to the expected value of 355 g/mol. This initial investigation of poly**1** thus supported our hypothesis that styrenic CDIs are a straightforward platform for PPM.

Copolymerization of functional monomers is frequently used to control the loading of reactive repeat units.^2,28^ Copolymerization of **1** and styrene resulted in poly(**1**-*co*-styrene)_5_ and poly(**1**-*co*-styrene)_10_, where the subscript indicates the mol % of **1** versus styrene in the initial monomer feed (Figure 1a). This loading was approximately maintained in the resulting copolymers (Figures S9 and S10), and all other characterizations were consistent with successful copolymerization (Figures S11–S14). To explore the scope of PPM, we combined poly(**1**-*co*-styrene)_10_ with a variety of amines (Figures 1b and S15–S30). As in the homopolymer, nucleophilic addition was usually complete in under 1 h with stoichiometrically equivalent amounts of amine and CDI functionalities, as evidenced by the disappearance of the CDI stretching frequency at ∼2100 cm^–1^ in the FT-IR spectrum. In most cases, product isolation was accomplished by removing the solvent *in vacuo*, and isolated yields were thus >95%. However, use of hydrochloride salts required the addition of triethylamine to form the desired nucleophilic species. This necessitated a precipitation to remove the triethylammonium byproduct, reducing the isolated yield. Similarly, sterically demanding amines such as 2-methylpiperidine required excess amine (1.5 equiv) for full CDI conversion, leading to lower yields due to the precipitation required for isolation. The reaction was tolerant of various functionalities, including alkenes, esters, and other potentially competing nucleophiles such as alcohols. To underscore the lack of reactivity of alcohols in this context, we combined benzyl alcohol and poly**1** and monitored the reaction over multiple days, during which no conversion was evident (Figure S31).

**Figure 1:**
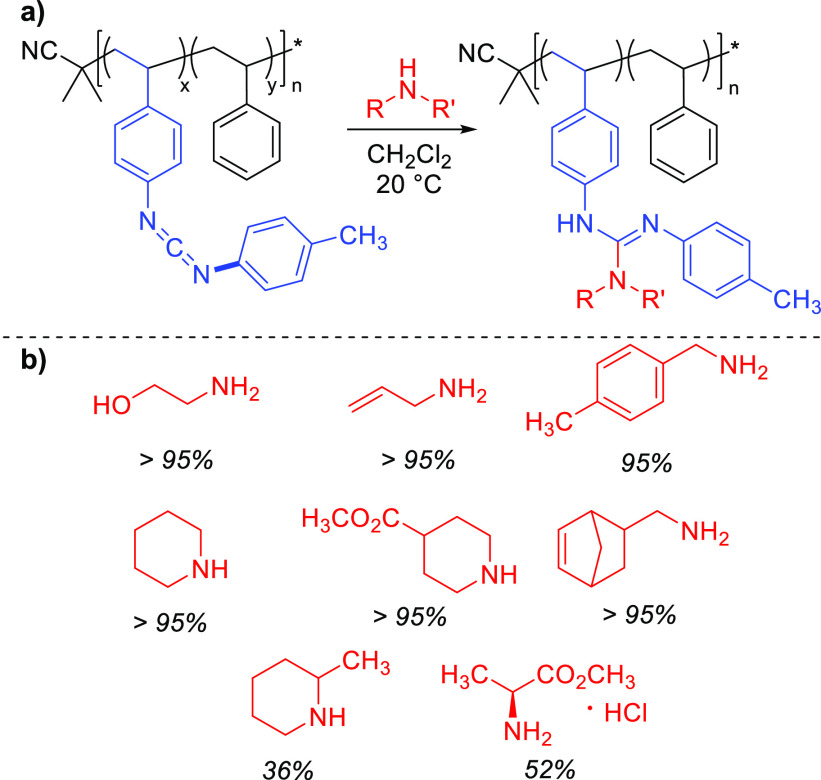
(a) Nucleophilic addition of primary and secondary alkyl amines to poly(**1**-*co*-styrene)_10_. R = alkyl; R′ = alkyl, H. (b) Amines used in this study. Isolated yields of modified polymer are given below each structure.

Reversible addition–fragmentation chain transfer (RAFT) copolymerization provides control of molar mass, end groups, and dispersity, which can be essential in preparing more complex polymeric architectures. To this end, we subjected **1** to RAFT copolymerization with styrene, using 2-cyano-2-propyl dodecyl trithiocarbonate as a chain transfer agent (CTA). SEC analysis indicated that the polymer possessed lower dispersity compared to poly(**1**-*co*-styrene)_10_ synthesized by free radical polymerization, and varying the ratio of monomer to CTA led to control of the molar mass (Figure 2). Additionally, the trithiocarbonate end group facilitates the synthesis of block polymers. When isolated poly(**1**-*co*-styrene)_10_ derived from RAFT polymerization was reinitiated in the presence of additional styrene, a shift to higher molar mass was observed in the SEC chromatogram, consistent with block polymer formation (Figure S32).

**Figure 2:**
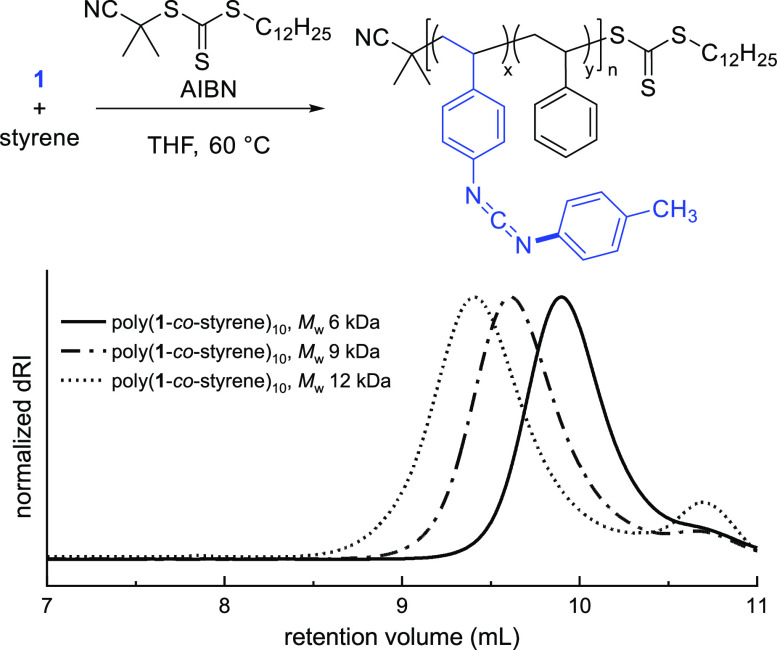
RAFT copolymerization of **1** and styrene. Chromatograms are of RAFT copolymers with varied total monomer/CTA ratios (99:1, 198:1, and 297:1, respectively); *Đ* ∼ 1.2 in each case. The peak at 10.8 mL retention volume is associated with solvent breakthrough.

Previous efforts in our group^29^ established that the reaction of multifunctional aryl CDIs with multifunctional amines results in formation of covalent adaptable networks (CANs),^30,31^ with thermal guanidine metathesis (TGM) as the operative mechanism of cross-link exchange.^25^ Poly(**1**-*co*-styrene)_5_ and poly(**1**-*co*-styrene)_10_ synthesized by free-radical copolymerization were subjected to guanylation with piperazine, resulting in cross-linking to yield CAN_5_ and CAN_10_, respectively (Figure 3a); we also included 5 wt % dioctyl phthalate as plasticizer to lower *T*_g_. To investigate the effects of the guanidine molecular structure, we reacted poly(**1**-*co*-styrene)_5_ with *trans*-2,5-dimethylpiperazine to yield a more sterically congested analogue, dm-CAN_5_. As recent studies have implicated prepolymer chain length as an important factor in determining CAN dynamics,^32^ we used poly(**1**-*co*-styrene)_5_ and poly(**1**-*co*-styrene)_10_ of similar molar masses and dispersities (*M*_w_ 14–18 kDa, *Đ* ∼ 1.5) for all our CAN investigations. Similar to modification reactions with monofunctional amines, the FT-IR spectrum of each CAN showed complete disappearance of the CDI stretching frequency (Figures S33–S35). DSC analysis indicated that *T*_g_ varied with the cross-link density and molecular structure (CAN_5_*T*_g_ 103 °C, CAN_10_*T*_g_ 108 °C, dm-CAN_5_*T*_g_ 91 °C; Figure S36), and TGA showed modest thermal stability with *T*_d,5%_ of 220–240 °C (Figure S37). Gel fractions of 85–90% in each case further supported successful network formation.

**Figure 3:**
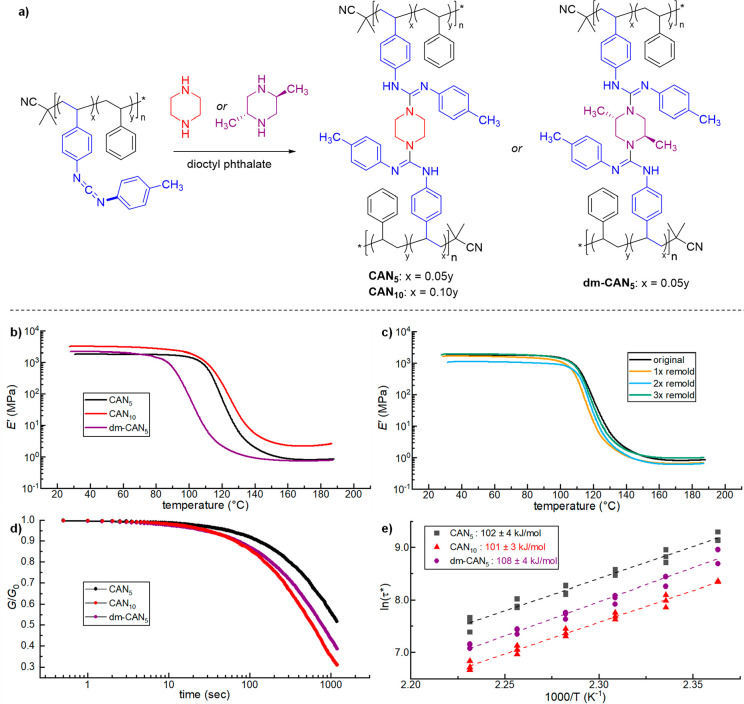
(a) Preparation of CAN_5_ and dm-CAN_5_ from poly(**1**-*co*-styrene)_5_ and CAN_10_ from poly(**1**-*co*-styrene)_10_. (b) DMA thermograms of *E*′ vs temperature for CAN_5_, CAN_10_, and dm-CAN_5_. (c) DMA thermograms of *E*′ versus temperature upon reprocessing a sample of CAN_5_ up to three times. (d) Normalized stress relaxation data for samples of CAN_5_, CAN_10_, and dm-CAN_5_ at 170 °C. (e) Arrhenius plot of CAN_5_, CAN_10_, and dm-CAN_5_. Relaxation times were extracted by fitting non-normalized stress relaxation data to a stretched exponential function. ln(τ*) from each experiment is shown on the plot; three individual samples of each CAN were used to construct the plot. *E*_a_ and standard error were calculated from each dashed line of best fit.

A hallmark feature of CANs is their ability to be (re)processed into homogeneous samples despite their network structure, which is enabled by dynamic cross-link exchange reactions that occur under specific conditions. Finely ground samples of each CAN were melt processed in a hydraulic press at 150 °C to yield specimens for rheological analysis. In each case, DMA temperature ramps showed a single drop in storage modulus (*E*′) corresponding to *T*_g_ along with a relatively constant *E*′ in the rubbery plateau (Figures 3b and S38 and Table S1). The plateau moduli of CAN_5_ and dm-CAN_5_ were similar (0.82 and 0.74 MPa at 170 °C, respectively), indicative of similar cross-link densities. Accordingly, the plateau modulus of the more highly cross-linked CAN_10_ (2.2 MPa at 170 °C) was greater than either CAN_5_ variant. Despite the dissociative nature of the TGM reaction,^25^ no significant drop in *E*′ was observed at higher temperatures. This is consistent with dissociative CAN systems in which *K*_eq_ greatly favors cross-link association at all experimental temperatures.^31,29^ Reprocessing experiments in which a sample of CAN_5_ was reground and subjected to melt pressing indicated that the material could be recycled multiple times, although some variation in *E*′ and *T*_g_ was observed with each cycle (Figures 3c and S39 and Table S2).

Stress relaxation studies of CANs lend insight into the molecular-level processes responsible for their dynamic behavior.^33,34^ Samples of CAN_5_, CAN_10_, and dm-CAN_5_ were each subjected to stress relaxation experiments in 5 °C increments from 150–175 °C in a parallel-plate shear rheometer. Characteristic relaxation times (τ*) at each temperature were obtained by fitting the non-normalized data to a stretched exponential,^35,36^ which can account for multiple modes of relaxation (Table S3 and Figures S40–S42). Relaxation times of dm-CAN_5_ were faster at all temperatures than that of CAN_5_: for example, τ* of dm-CAN_5_ at 170 °C (∼1650 s) was approximately 65% that of CAN_5_ at the same temperature (∼2537 s; Figure 3d). This is consistent with the effects of sterics on TGM reaction kinetics, as more sterically congested guanidines undergo more rapid exchange.^25^ The more highly cross-linked CAN_10_ also possessed shorter τ* (∼1150 s at 170 °C) compared to CAN_5_ at all temperatures. This can be rationalized as an effect of the concentration of reactive species: as CAN_10_ possesses more guanidine functionalities per chain, it is easier for a dissociated reactive pair to find new partners to rearrange the network and relax stress. Studies on other dissociative systems have found similar trends,^37,38^ though the situation appears to be more complex in associative CANs.^39,40^

Each material exhibited an Arrhenius scaling of τ* with temperature (Figure 3e), another characteristic feature of CANs. As expected for materials in which the same cross-link exchange reaction was operative, CAN_5_ and CAN_10_ possessed similar activation energies (*E*_a_ = 101 and 102 kJ/mol, respectively). Thus, it appears the difference in τ* in these systems can be attributed to the pre-exponential factor in the Arrhenius equation, as evidenced by the changes in y-intercept in the Arrhenius plot.^38,41^ While *E*_a_ calculated in an analogous small molecule model system was lower (72 kJ/mol; Figure S43 and Table S4), this is consistent with previous experimental observations^29,42,43^ and theoretical predictions.^44,45^ Broadly, the difference is attributed to the additional energy required for network strand diffusion and relaxation, which is not present in small molecules. Interestingly, dm-CAN_5_ displayed a similar *E*_a_ (108 kJ/mol) as CAN_5_ and CAN_10_, despite its small molecule analogue having a lower *E*_a_ (56 kJ/mol). Though the nature of this discrepancy is not clear from these preliminary studies, it further emphasizes the important and complex role of the polymer matrix and pre-exponential factor in determining CAN dynamics.

In conclusion, we have established the nucleophilic addition of alkyl amines to aryl carbodiimides as a general postpolymerization modification strategy. Homo- and copolymerization of a carbodiimide-containing styrenic monomer result in reactive polymers that can be functionalized under ambient conditions with no additives, resulting in simple purification and high yields of modified polymer. Further, we used this platform to prepare CAN materials with varied cross-link density and guanidine structure, finding that the relaxation times of these materials varied with each factor while the activation energies were largely similar. We hope this will stimulate additional interest in the uses of the carbodiimide moiety in polymeric contexts and that it further emphasizes the role of the polymer matrix and pre-exponential factor in impacting CAN rheology. To this end, we aim to conduct detailed studies of the various intersecting factors impacting the dynamics of guanidine-based CANs and adapt the carbodiimide functionality to other types of polymeric materials.

## Supporting Information Available

Experimental details for synthesis of small molecules and polymers; additional characterization of polymers, modified copolymers, and CANs; data from DMA and stress relaxation analyses of CANs; and ^1^H and ^13^C NMR spectra of **1** and intermediates (PDF)
