# Supplementary material for: Postpolymerization Modification by Nucleophilic Addition to Styrenic Carbodiimides
Source: ACS Macro Lett. 2023 Jul 24;12(8):1112–7. doi: 10.1021/acsmacrolett.3c00382 (PMC10433525; doi:10.1021/acsmacrolett.3c00382)
Supplement: Supplementary file 1 — mz3c00382_si_001.pdf [file mz3c00382_si_001.pdf]

Supporting Information

Postpolymerization Modification by Nucleophilic Addition to Styrenic Carbodiimides

*Hayden E. Houck,[†] Kate A. McConnell,[†] Conner J. Klingler, Adelle L. Koenig, Grace K. Himka, and Michael B. Larsen**

[†]These authors contributed equally.

*Corresponding author: mike.larsen@wwu.edu

Department of Chemistry, Western Washington University, Bellingham, WA, USA 98225

Materials. Unless otherwise noted, all chemicals were purchased from commercial sources and used as received. Dry tetrahydrofuran (THF) and dichloromethane (CH_2Cl_2) were obtained from an Inert PureSolv solvent purification system. Triethylamine was dried over 4 Å molecular sieves and distilled under nitrogen.

Instrumentation. ^{1}H and ^{13}C NMR spectra were recorded on a Bruker Avance III 500 MHz FT-NMR spectrometer. Chemical shifts are reported in delta (δ) units, expressed in parts per million (ppm) downfield from tetramethylsilane using the residual protio-solvent as an internal standard ($CDCl_3$, ^{1}H: 7.26 ppm and ^{13}C: 77.16 ppm). SEC analysis was performed in THF (1.0 mL/min) on a Malvern Viscotek GPCMax equipped with Phenomenex Phenogel 5 μm 10^4 Å column, a SEC-MALS 9 multiangle light scattering detector, viscometer, and differential refractive index detector. MS analysis of small molecules was performed with an Agilent 6545XT LC/Q-TOF spectrometer in positive polarity. MALDI-MS analysis was performed with a Bruker autoflex maX MALDI-TOF/TOF spectrometer using *trans*-2-[3-(4-*tert*-butylphenyl)-2-methyl-2-propenylidene] malonitrile (DCTB) as matrix for poly**1** or 2,5-dihydroxybenzoic acid (DHB) for the modified poly**1**. Samples were prepared by mixing matrix solution (DCTB or DHB, 25 mg/mL in THF) with polymer solution (2 mg/mL in THF) and silver trifluoroacetate (5 mg/mL in THF) at a ratio of 100:15:1. IR spectroscopy was performed with a Thermo iS10 FT-IR with single-bounce diamond ATR. Thermogravimetric analyses (TGA) were performed on a TA Instruments Q500 with a platinum pan. Ramp experiments were heated at 20 °C/min from room temperature to 550 °C or

600 °C under nitrogen flow (40 mL/min). Differential scanning calorimetry (DSC) experiments were performed in aluminum sample pans. Ramp experiments were heated at 15 °C/min from ambient temperature to 180 °C, cooled at 15 °C/min from 180 °C to 0 °C, and heated at 15 °C/min from 0 °C to 180 °C with 5 min isotherms at each extreme under nitrogen flow (50 mL/min). Dynamic mechanical analyses (DMA) were performed on a TA Instruments Q800 in tension mode. For temperature ramp experiments, samples were deformed by a 0.025% sinusoidal tensile strain. Each rectangular-shaped sample (approximately 30 mm × 5 mm × 2.5 mm) was heated from ambient conditions to 190 °C at a rate of 3 °C/min. All experiments were conducted at a frequency of 1 Hz and the glass transition temperature (T_g) was taken as the peak of tan δ. Stress relaxation experiments were performed on a TA Instruments DHR-2 Discovery Hybrid Rheometer using a 25 mm parallel plate geometry. After being held at each set temperature for 60 sec, samples were deformed by 1% strain for 1200 sec; this strain was determined to lie within the linear viscoelastic region for all materials tested.

Scheme S1. Synthesis of **1**, poly**1**, and poly(**1**-*co*-styrene).

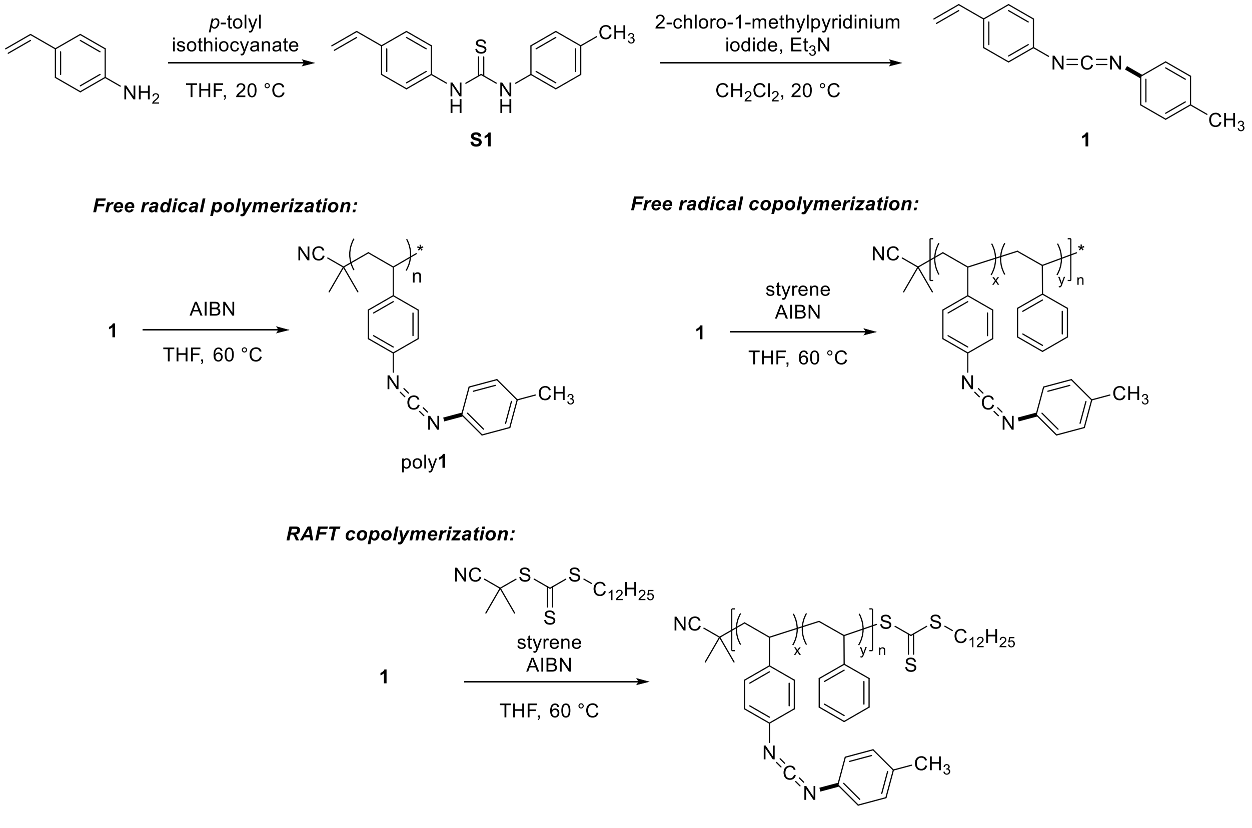

*Preparation of thiourea **S1***: 4-aminostyrene (5.00 mL, 42.7 mmol, 1.0 equiv.) was added to a flask under N_2 containing dry THF (30 mL). To this solution, *p*-tolyl isothiocyanate (6.69 g, 44.8 mmol, 1.05 equiv.) was added and the reaction mixture was stirred for 2 h while monitoring via TLC (2:1 v/v hexanes/ethyl acetate). Upon full conversion of 4-aminostyrene, hexanes (20 mL) was added to precipitate the crude product. The precipitate was recovered by gravity filtration and air dried overnight to yield thiourea **S1** as a white crystalline solid (8.69 g, 76%). ^{1}H NMR (500 MHz, $CDCl_3$) δ 7.79 (bs, 1H), 7.74 (bs, 1H), 7.43 (d, *J* = 8.6 Hz, 2H), 7.38 (d, *J* = 8.5 Hz, 2H), 7.27 – 7.18 (m, 4H), 6.71 (dd, *J* = 17.6, 10.9 Hz, 1H), 5.75 (d, *J* = 17.6 Hz, 1H), 5.29 (d, *J* = 10.8 Hz, 1H), 2.39 (s, 3H). ^{13}C NMR (126 MHz, $CDCl_3$) δ 180.0, 137.4, 136.8, 136.3, 135.9, 134.4, 130.4, 127.2, 125.6, 125.2, 114.6, 21.2. MS (ESI): $[M+H]^+$ calc'd for $C_{16}H_{17}N_2S$ 269.1112, found 269.1107.

*Preparation of **1***: In an oven-dried flask under N_2, thiourea **S1** (3.50 g, 13.0 mmol, 1.0 equiv.) and 2-chloro-1-methylpyridinium iodide (4.00 g, 15.7 mmol, 1.2 equiv.) were dissolved in dry dichloromethane (30 mL). Triethylamine (5.43 mL, 39.0 mmol, 3.0 equiv.) was added and the reaction was stirred at ambient temperature for 30 min. The reaction was monitored by TLC (2:1 v/v hexanes/ethyl acetate), and upon full conversion of **S1** (~30 min) the solvent was removed by rotary evaporation. The resulting residue was redissolved in 2:1 v/v hexanes/dichloromethane and purified by elution through a silica plug with the same solvent, yielding monomer **1** as a clear, slightly yellow oil (2.71 g, 89%). ^{1}H NMR (500 MHz, $CDCl_3$) δ 7.37 (d, *J* = 8.4 Hz, 2H), 7.19 – 7.12 (m, 4H), 7.08 (d, *J* = 8.3 Hz, 2H), 6.68 (dd, *J* = 17.6, 10.9 Hz, 1H), 5.71 (dd, *J* = 17.6, 0.8 Hz, 1H), 5.23 (dd, *J* = 10.8, 0.8 Hz, 1H), 2.33 (s, 3H). ^{13}C NMR (126 MHz, $CDCl_3$) δ 138.3, 136.1, 135.7, 135.6, 135.2, 130.3, 127.4, 124.4, 124.2, 113.8, 21.1. MS (ESI): calc'd for $C_{16}H_{15}N_2$ 235.1235, found 235.1233.

*General procedure for free-radical polymerization of **1***: In an oven-dried Schlenk flask under N_2, AIBN (0.0700 g, 0.427 mmol, 0.10 equiv.) was dissolved in dry THF (5.0 mL). Monomer **1** (1.00 g, 4.27 mmol, 1.0 equiv.) was added to the reaction flask, which was then subjected to three freeze-pump-thaw cycles. The headspace was backfilled with N_2 after the final thaw and the reaction heated to 60 °C; the pressure in the flask was then equalized. The reaction was stirred at 60 °C for 16 h, at which time the reaction was terminated by opening to air. The volume of the crude polymer

mixture was reduced by half via rotary evaporation and precipitated dropwise into chilled methanol + 1% v/v deionized water (~10× the reduced reaction mixture volume). The precipitated polymer was then collected by vacuum filtration and redissolved in minimal THF. A second precipitation was performed using chilled hexanes (~10× the volume of the redissolved solution). The precipitate was collected by vacuum filtration and the remaining solvent was removed under vacuum at 60 °C to yield poly**1** as a white powder (0.425 g, 43%).

*General procedure for copolymerization of **1** and styrene*: To an oven-dried 250 mL Schlenk flask containing dry THF (40 mL) was added **1** (2.60 g, 11.1 mmol, 1.0 equiv.), styrene (23.12 mL, 221.9 mmol, 20 equiv.), and AIBN (2.02 g, 12.3 mmol, 1.1 equiv.; 5.5 mol% versus total monomer concentration). The reaction flask was then sealed and the reaction mixture subjected to three freeze-pump-thaw cycles. The headspace was backfilled with N_2 after the final thaw and the reaction mixture was stirred at 60 °C for 16 hours, at which time the vessel was opened to air to terminate the reaction. The crude polymer mixture was precipitated dropwise into chilled methanol + 1% deionized water (400 mL). The precipitated polymer was recovered via vacuum filtration, redissolved in THF (40 mL), re-precipitated dropwise into an additional 400 mL methanol, and filtered once more. The polymer was dried on a Schlenk line on high vacuum at 40 °C to give poly(**1**-*co*-styrene)$_5$ as a white powder (16.0 g, 70%). Loading of CDI functionality per g of poly(**1**-*co*-styrene) was first determined using ^{1}H NMR spectroscopy with an internal standard. Typical values were 0.4 – 0.6 mmol CDI / g poly(**1**-*co*-styrene)$_5$ and 0.8 – 1.0 mmol CDI / g poly(**1**-*co*-styrene)$_{10}$.

*General procedure for RAFT copolymerization of **1** and styrene*: In an oven-dried Schlenk flask under N_2, 2-cyano-2-propyl dodecyl trithiocarbonate (0.041 g, 0.12 mmol, 1.0 equiv.) was dissolved in dry THF (2.0 mL). AIBN (5.8 mg, 0.035 mmol, 0.3 equiv.), styrene (1.23 mL, 10.67 mmol, 90 equiv.) and **1** (0.25 g, 1.07 mmol, 9.0 equiv.) were then added to the reaction flask. The reaction flask was then sealed and the reaction mixture subjected to three freeze-pump-thaw cycles. The headspace was backfilled with N_2 after the final thaw and the reaction mixture was stirred at 60 °C for 16 hours, at which time the vessel was opened to air to terminate the reaction. The volume of the crude polymer mixture was reduced by half via rotary evaporation and precipitated dropwise into chilled methanol + 1% deionized water (~10× the reduced reaction

mixture volume). The precipitated polymer was then collected by vacuum filtration and redissolved in minimal THF. The second precipitation was performed using chilled hexanes (~10× the volume of the redissolved solution). The precipitate was collected by vacuum filtration and the remaining solvent was removed under vacuum at 60 °C. Variation in total monomer:CTA ratios were accomplished by adjusting the amount of CTA added; ratios examined were 99:1 (above), 198:1, and 297:1.

*General procedure for modification of poly(**1**-co-styrene)$_{10}$:* In a 20 mL scintillation vial, poly(**1**-*co*-styrene)$_{10}$ (0.100 g, 0.083 mmol CDI repeat unit, 1.0 equiv.) was dissolved in 0.4 mL CH_2Cl_2. The desired amine was added (0.083 mmol, 1.0 equiv.) and the reaction stirred under ambient conditions. After 1 h, sample was analyzed by ATR FT-IR spectroscopy to confirm full disappearance of the CDI stretching frequency at ~2100 cm^{-1}. The modified copolymer was then isolated by removing volatiles under vacuum at 60 °C. In the case of 2-methylpiperidine, 1.5 equiv. of amine was used, and polymer isolation was accomplished by precipitating the solution in hexanes (10 mL) followed by filtration. In the case of *L*-alanine methyl ester hydrochloride, 1.5 equiv. of amine was used; additionally, 1.0 equiv. of triethylamine was added to the reaction solution. The modified polymer was isolated by precipitation in hexanes, followed by filtration and subsequent trituration with water to remove residual hydrochloride salts.

General procedure for CAN synthesis: Poly(**1**-*co*-styrene)$_5$ (5.00 g, 2.97 mmol CDI repeat unit, 1.95 equiv.) was dissolved in 20 mL THF. In a separate vial, piperazine (0.131 g, 1.52 mmol, 1.0 equiv.) and dioctyl phthalate (0.265 mL, 5% w/w total reagent mass) were dissolved in 10 mL THF. The solutions were combined, rapidly stirred together, and poured into a glass dish at ambient temperature. The dish was left for 24 h for solvent evaporation to occur. The solid product was removed from the glass dish, broken into smaller pieces, and dried at 60 °C overnight on a high vacuum Schlenk line. The product was then crushed into a fine powder with a mortar and pestle and placed in a vacuum oven for 2 h at 100 °C to yield CAN$_5$ as a light yellow powder (4.80 g, 89%). For synthesis of CAN$_{10}$, poly(**1**-*co*-styrene)$_{10}$ was used and the amount of piperazine adjusted to maintain the same molar ratio as above. For dm-CAN$_5$, *trans*-2,5-dimethylpiperazine was used in place of piperazine while maintaining the same molar ratio as above.

General procedure for preparation of CAN samples: Approximately 500 mg of CAN was ground into a fine powder with a mortar and pestle and put in 25 mm dry pressing die set (MSE Supplies PR0104). The die was heated in a benchtop Carver press at 150 °C for 20 minutes with no pressure. 5000 psi was then applied for 20 min while maintaining heating. Following this, the die was moved to the benchtop and cooled for 20 min at ambient temperature, after which the sample disc was extracted from the mold. For samples used in DMA, approximately 500 mg of ground CAN was placed in a rectangular (30 mm × 5 mm × 2.5 mm) aluminum mold with aluminum plates on either side. The mold was placed in a benchtop Carver press at 150 °C and 5000 psi was immediately applied. The pressure and heat were maintained for 40 min. The mold was then immediately moved to a hot plate preheated to 150 °C, and samples were pushed out from the mold using a preheated rectangular die.

Procedure for small molecule kinetics studies: Kinetics studies of guanidines **S2** and **S3** were conducted in triplicate at 130, 150, and 160 °C as previously described.[1]

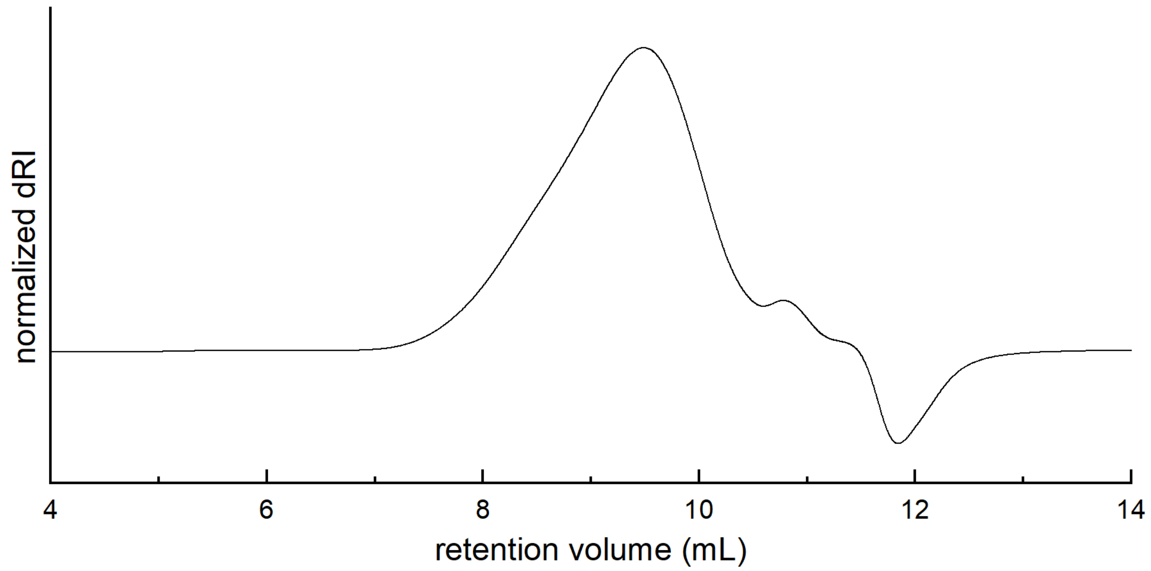

Figure S1. SEC chromatogram of poly**1** obtained by free radical polymerization. Peaks at 10.8 mL of retention volume and greater are associated with solvent breakthrough. M_w 129 kDa, *Đ* 3.5.

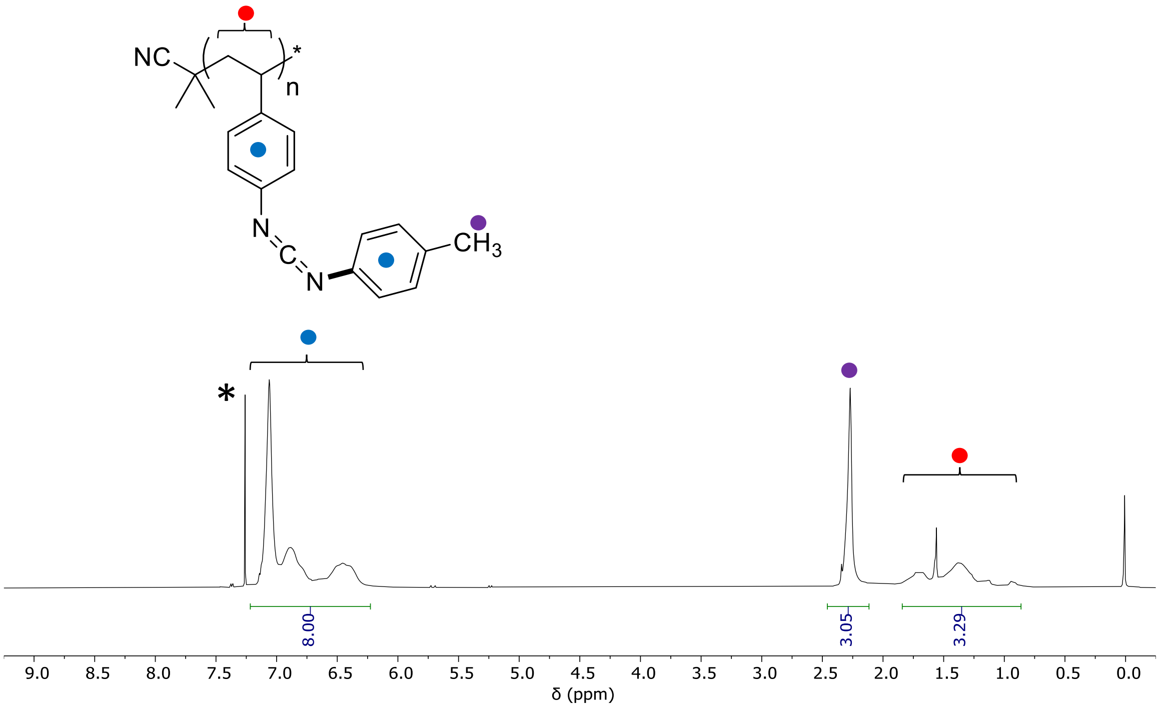

Figure S2. ^{1}H NMR spectrum (500 MHz, $CDCl_3$) and assignments of poly**1** synthesized by free radical polymerization. * = $CHCl_3$.

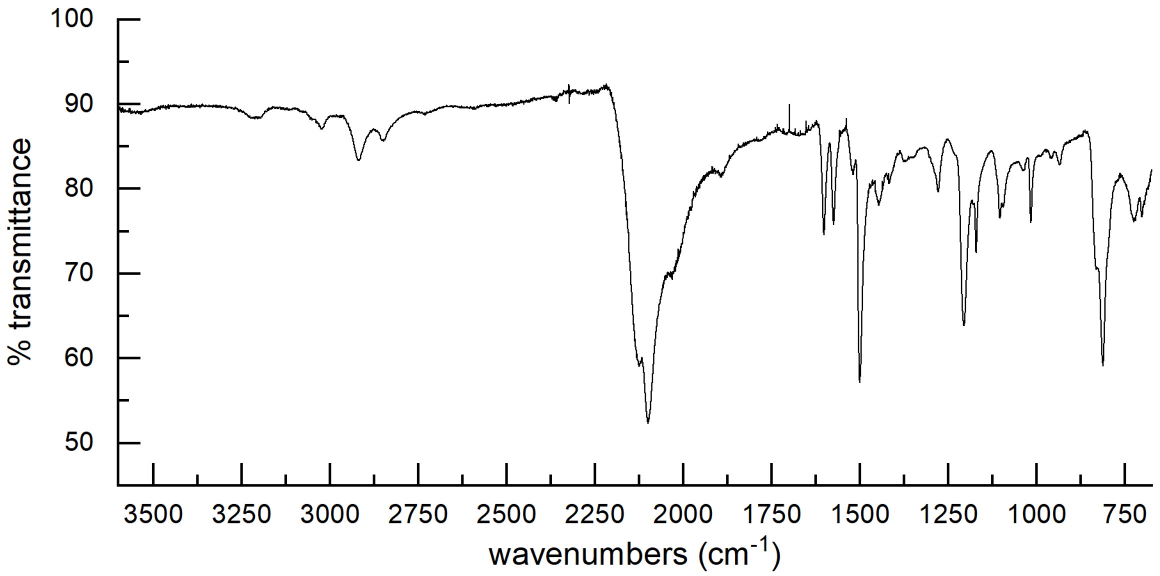

Figure S3. ATR FT-IR spectrum of poly**1**. Absorbance at ~2100 cm^{-1} attributed to stretching of the carbodiimide functionality.

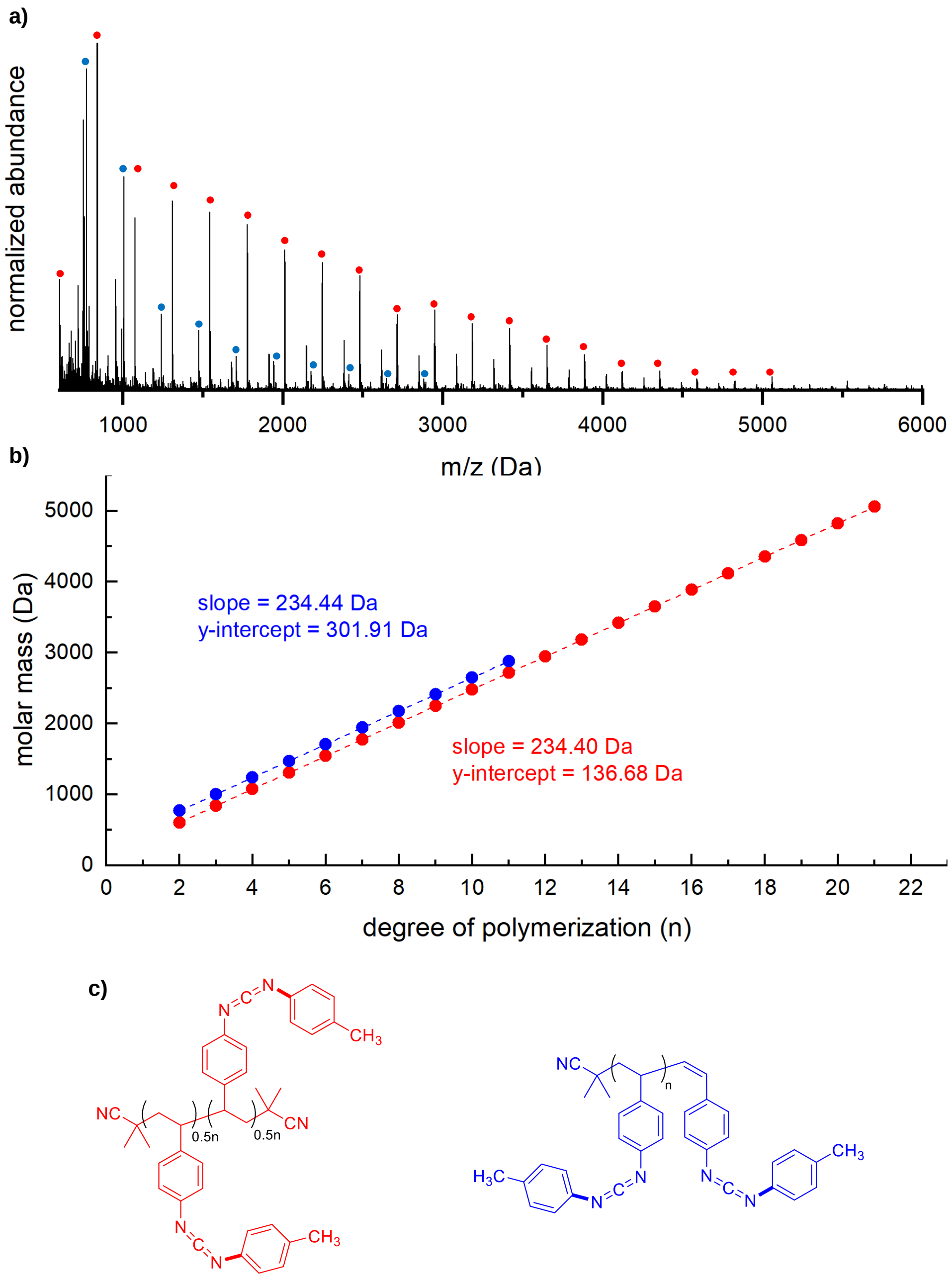

Figure S4. a) MALDI-MS spectrum (DCTB matrix, 600 – 6000 Da) of poly**1**. Indicated peaks are those used for analysis. b) Repeat unit and endgroup analysis of indicated populations.[2] Slope and y-intercept were calculated from the linear best fit (dashed lines). c) Structures assigned to populations indicated in MALDI-MS spectrum. Termination by recombination (red) and termination by disproportionation (blue) are both present; total endgroup masses correspond to y-intercepts in both populations.

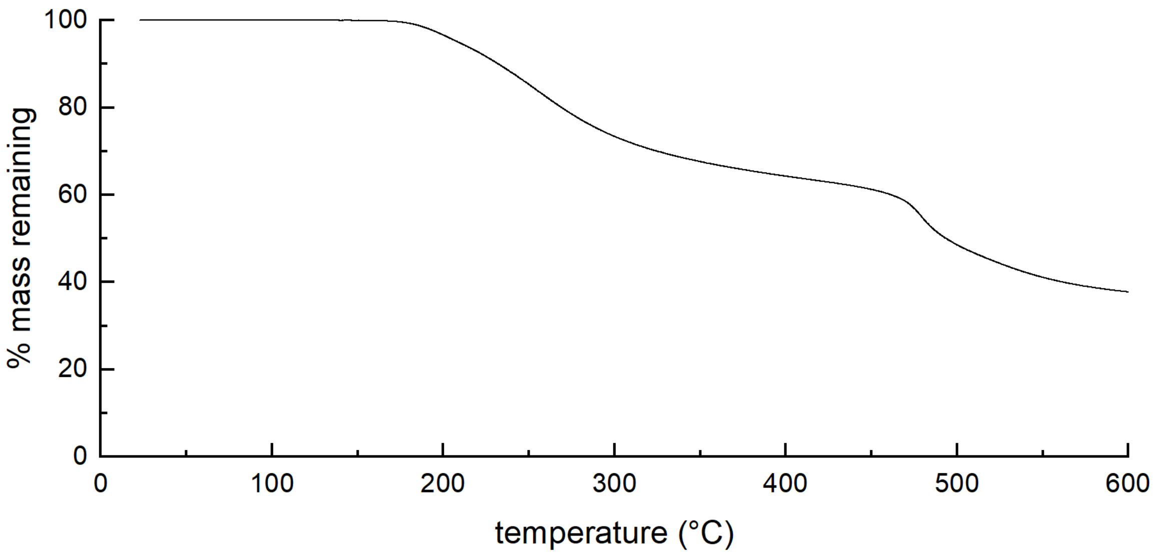

Figure S5. TGA thermogram (20 °C/min, N_2 atmosphere) of poly**1**. $T_{d,5\%}$ = 209 °C.

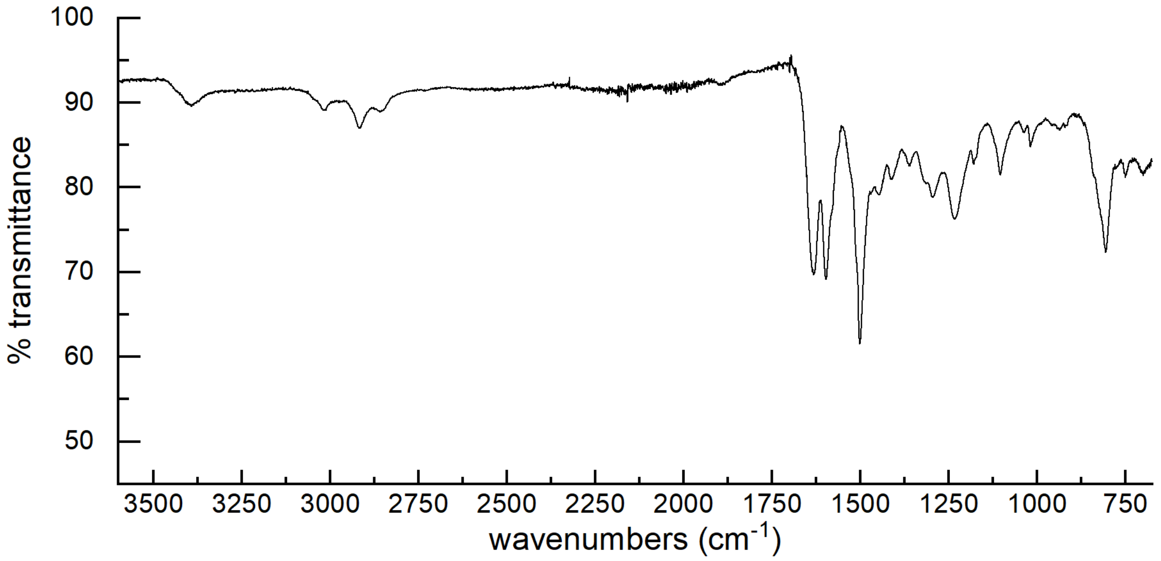

Figure S6. ATR FT-IR spectrum of poly**1** modified with 4-methylbenzyl amine. Note complete disappearance of absorbance attributed to CDI stretching frequency (~2100 cm^{-1}) and appearance of absorbance attributed to guanidine C=N stretching (~1630 cm^{-1}).

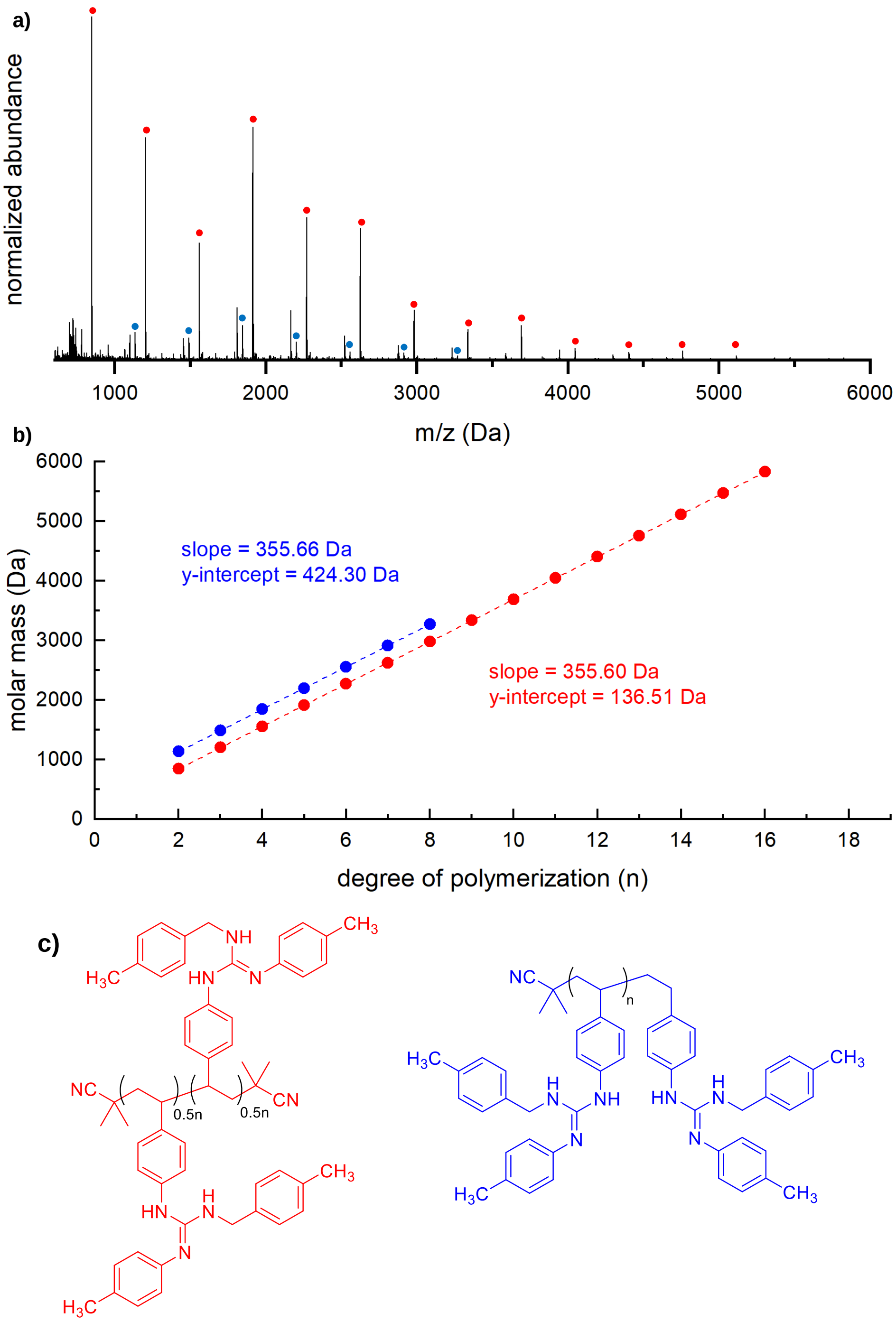

Figure S7. a) MALDI-MS spectrum (DHB matrix, 600 – 6000 Da) of poly**1** modified with 4-methylbenzyl amine. Indicated peaks are those used for analysis. b) Repeat unit and endgroup analysis of indicated populations.[2] Slope and y-intercept were calculated from the linear best fit (dashed lines). c) Structures assigned to populations indicated in MALDI-MS spectrum. Termination by recombination (red) and termination by disproportionation (blue) are both present; total endgroup masses correspond to y-intercepts in both populations.

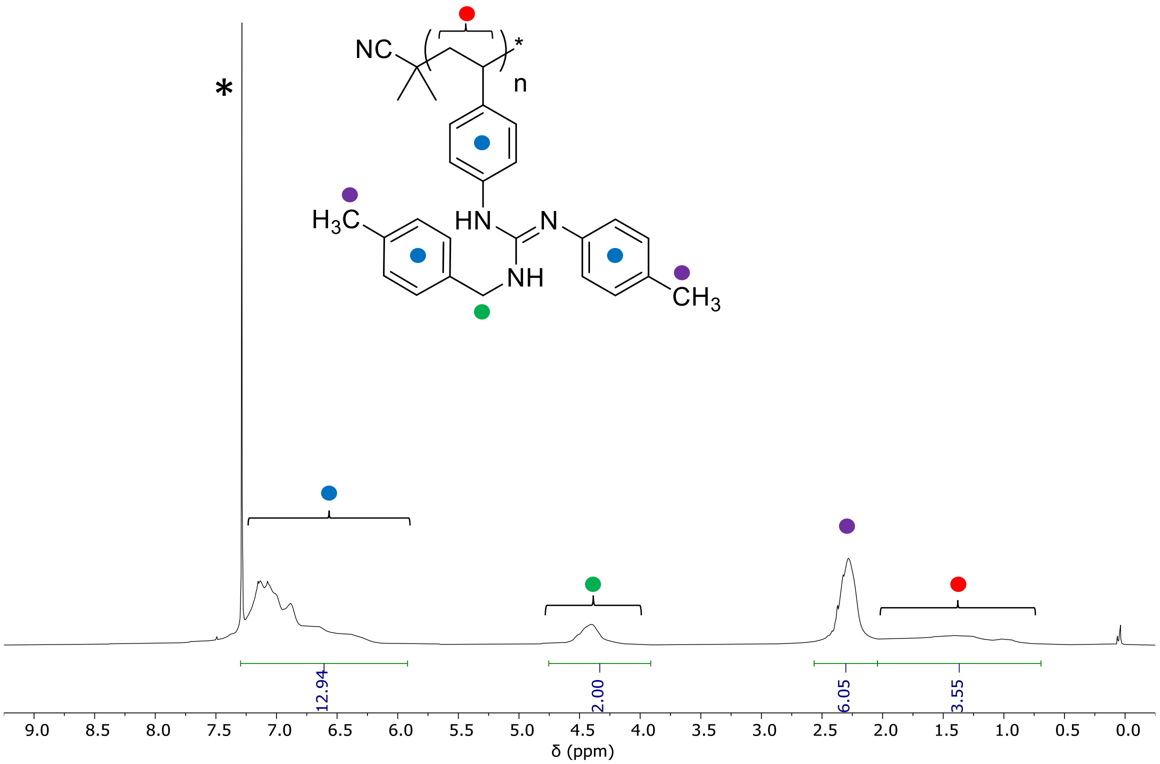

Figure S8. ^{1}H NMR spectrum (500 MHz, $CDCl_3$) and assignments of poly**1** modified with 4-methylbenzyl amine. * = $CHCl_3$.

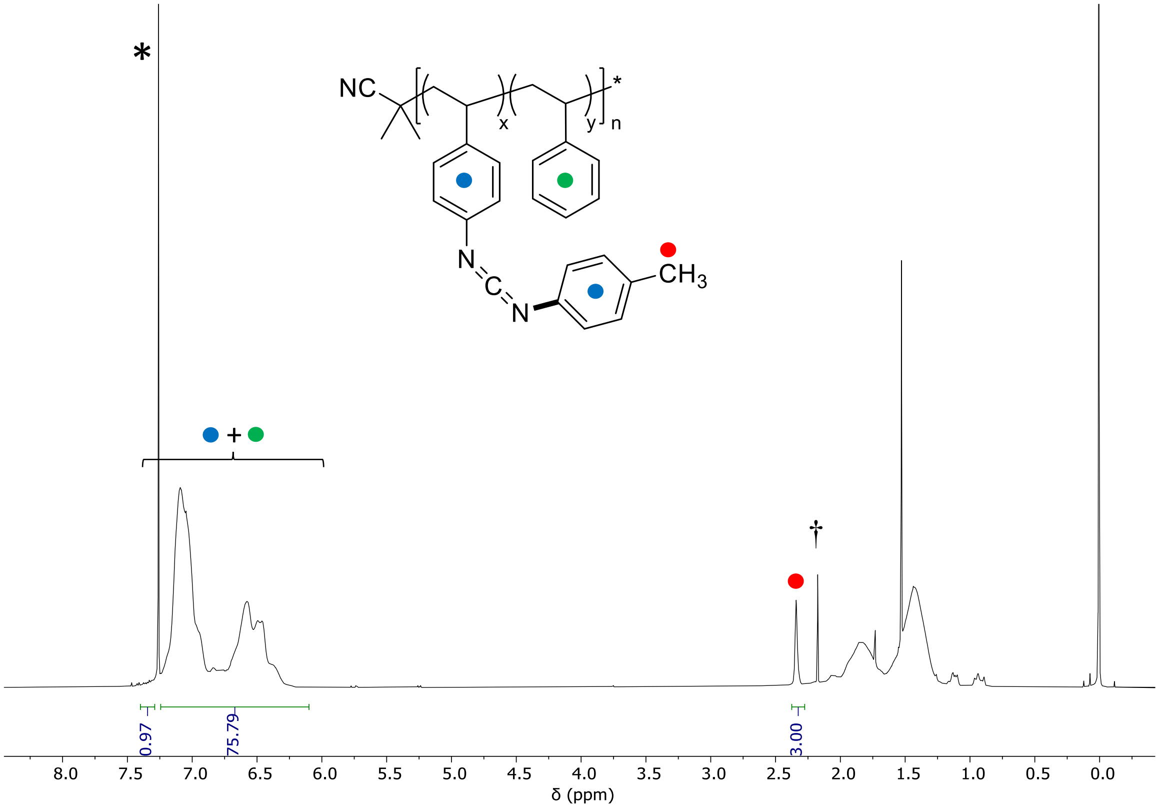

Figure S9. ^{1}H NMR spectrum (500 MHz, $CDCl_3$) and assignments of poly(**1**-*co*-styrene)$_5$. * = $CHCl_3$, † = acetone. Styrene:**1** ratio = 13.8:1, which equals 7.3 mol% **1** versus styrene in the obtained copolymer (see discussion below for calculation).

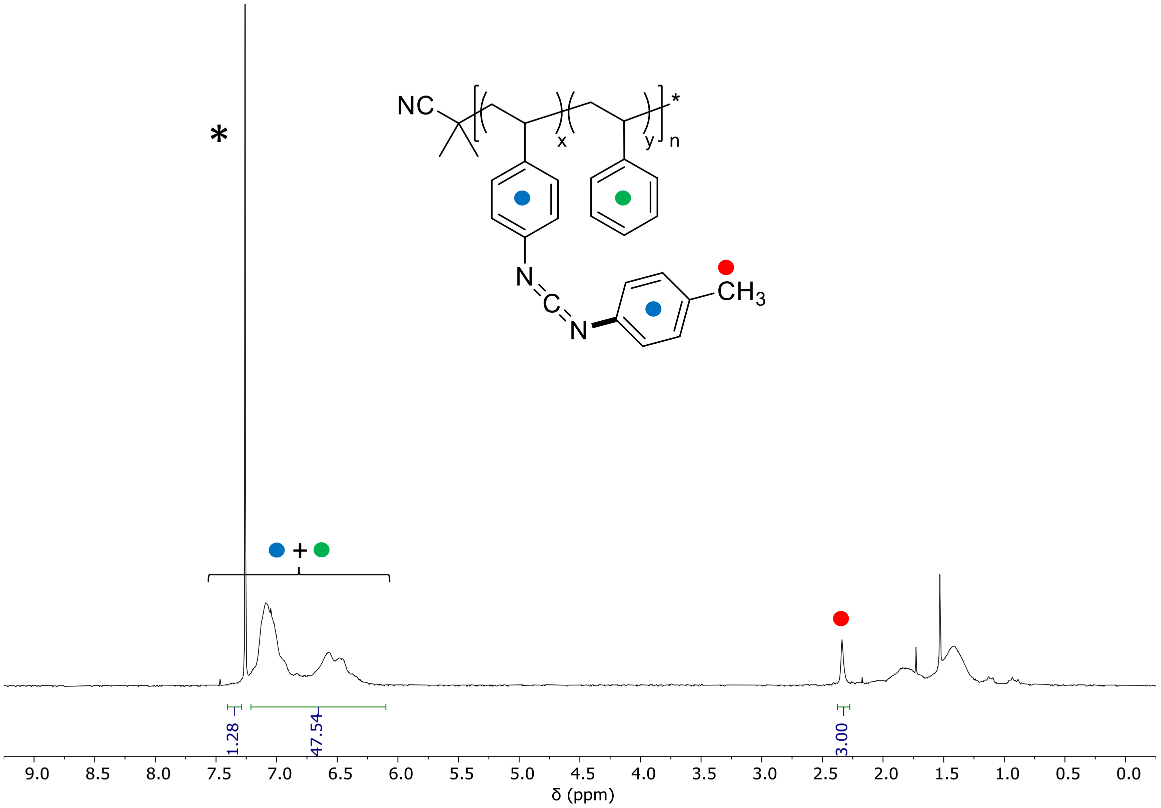

Figure S10. ^{1}H NMR spectrum (500 MHz, $CDCl_3$) and assignments of poly(**1**-*co*-styrene)$_{10}$. * = $CHCl_3$. Styrene:**1** ratio = 8.16:1, which equals 12.2 mol% **1** versus styrene in the obtained copolymer (see discussion below for calculation).

Calculation of copolymer composition.

The resonance at δ = 2.341 ppm is assigned to the aryl-CH_3 protons on each repeat unit derived from **1**. This signal is first integrated and normalized to 3.00. Integration of the entire aromatic region (excluding the residual $CHCl_3$ signal) encompasses the aromatic signals assigned to **1** and styrene. Subtraction of 8.00 from this integral removes the contribution of **1** to this value. The remaining value is attributed solely to styrene, which contains 5 protons per repeat unit. Thus, dividing the remaining value by 5 provides the number of styrene repeat units per **1**, the inverse of which is the mol% **1** versus styrene. Mathematically:

$$Styrene\ per\ \mathbf{1} = \frac{Aryl\ integration - 8}{5}$$

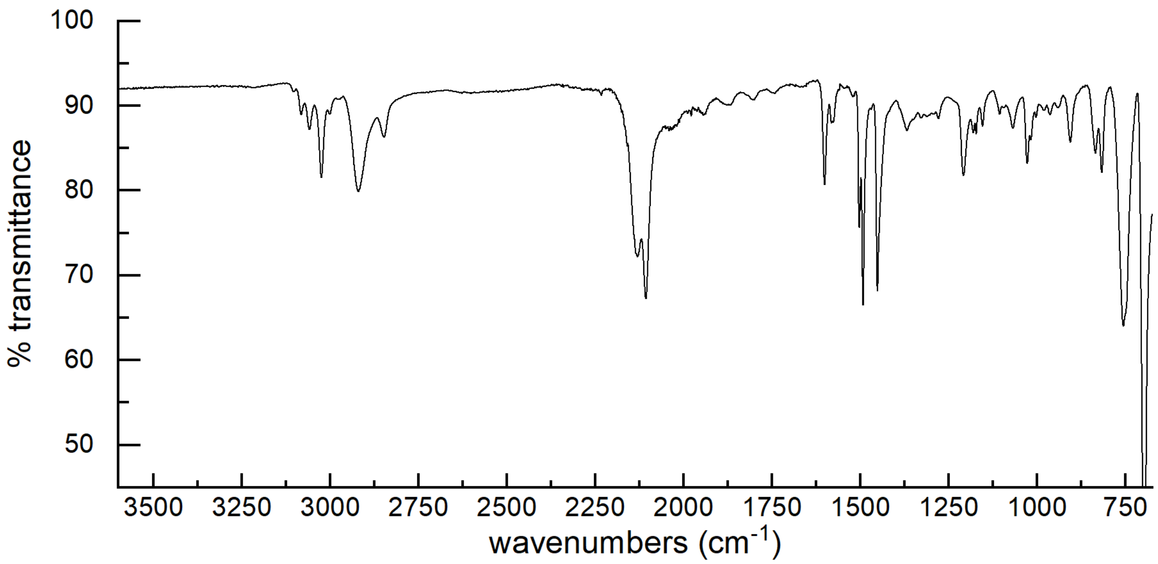

Figure S11. ATR FT-IR spectrum of poly(**1**-*co*-styrene)$_5$.

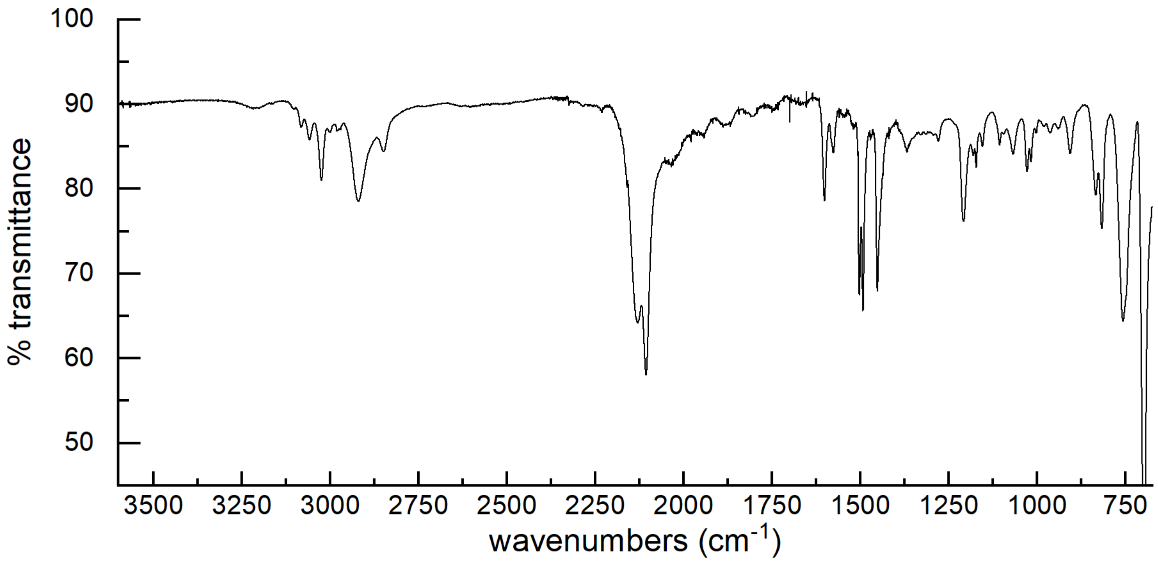

Figure S12. ATR FT-IR spectrum of poly(**1**-*co*-styrene)$_{10}$.

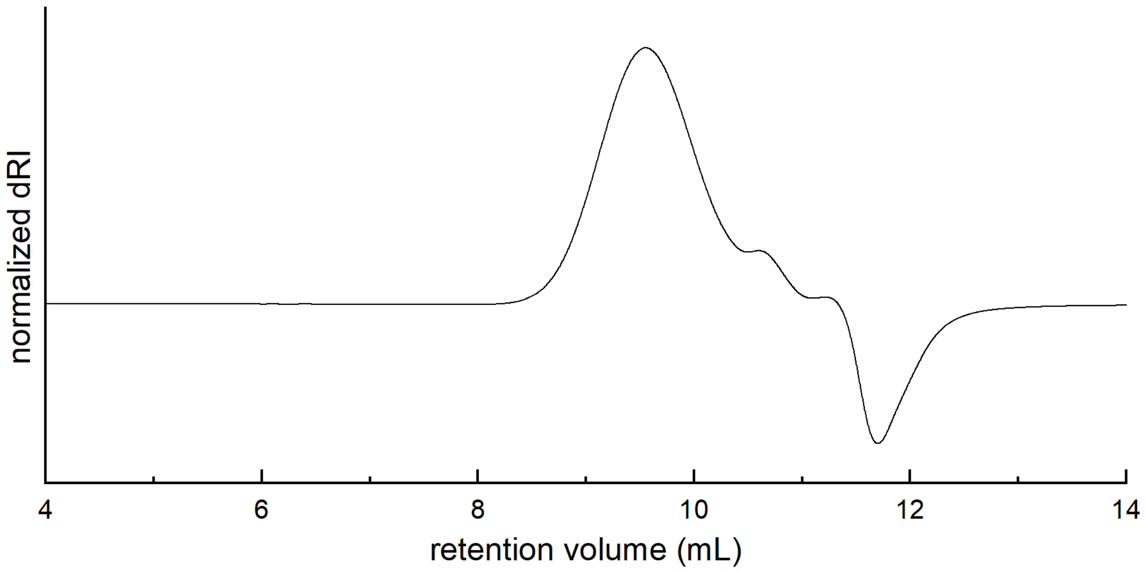

Figure S13. SEC chromatogram of poly(**1**-*co*-styrene)$_5$ obtained by free radical copolymerization. Peaks at 10.8 mL of retention volume and greater are associated with solvent breakthrough. M_w 14 kDa, M_n 9 kDa, *Đ* 1.5.

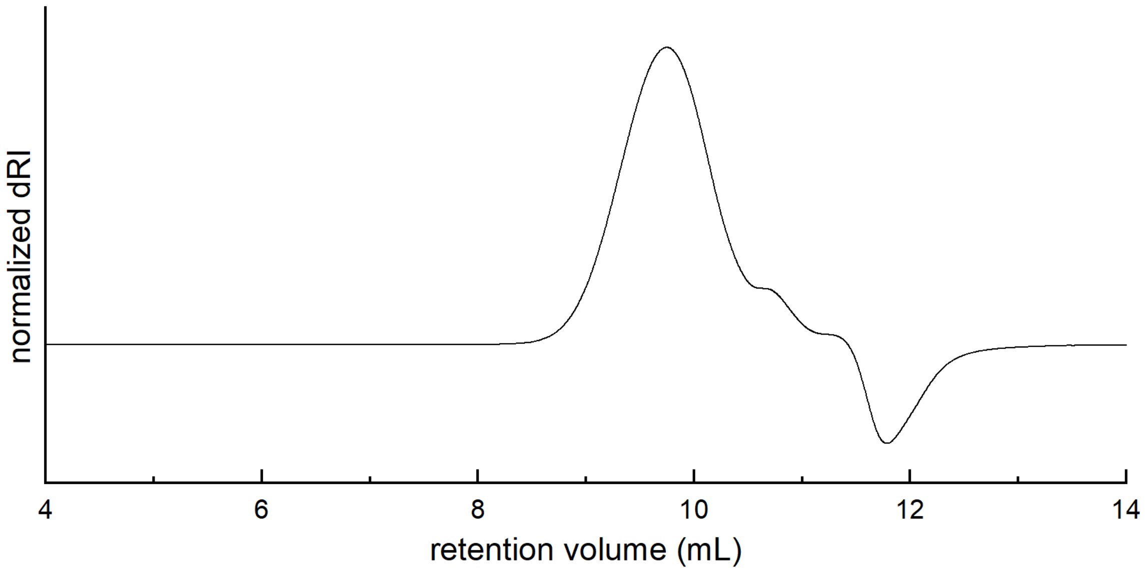

Figure S14. SEC chromatogram of poly(**1**-*co*-styrene)$_{10}$ obtained by free radical copolymerization. Peaks at 10.8 mL of retention volume and greater are associated with solvent breakthrough. M_w 17 kDa, M_n 11 kDa, *Đ* 1.5.

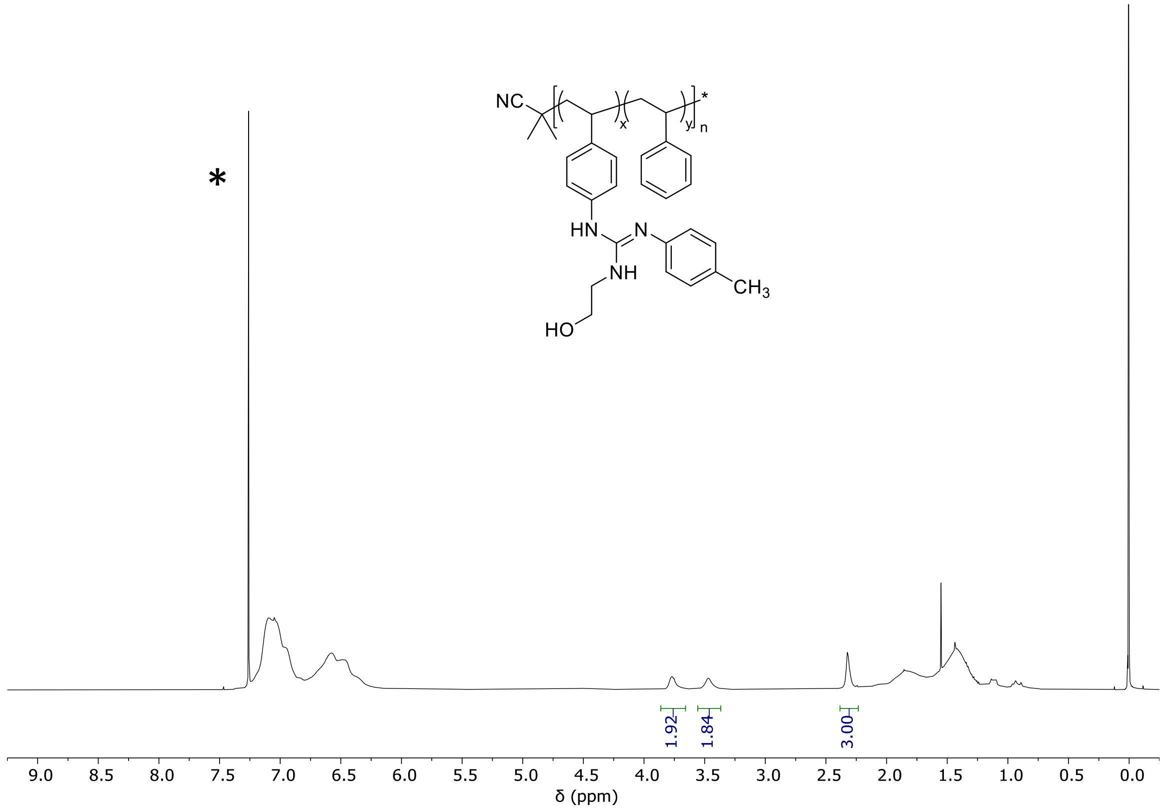

Figure S15. ^{1}H NMR spectrum (500 MHz, $CDCl_3$) and structure of poly(**1**-*co*-styrene)$_{10}$ modified with 2-aminoethanol. * = $CHCl_3$.

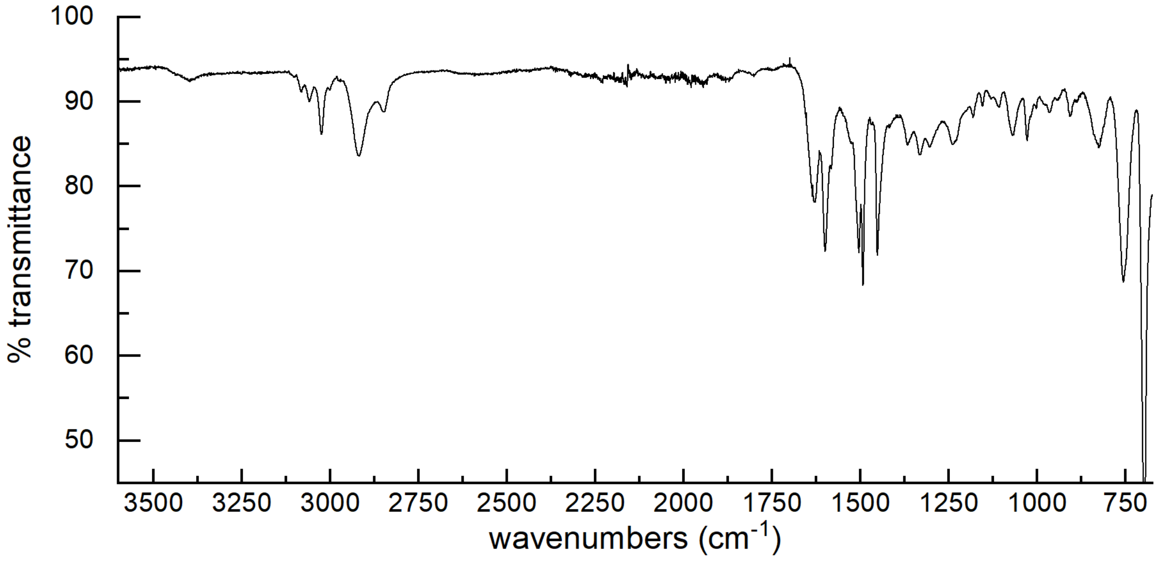

Figure S16. ATR FT-IR spectrum of poly(**1**-*co*-styrene)$_{10}$ modified with 2-aminoethanol.

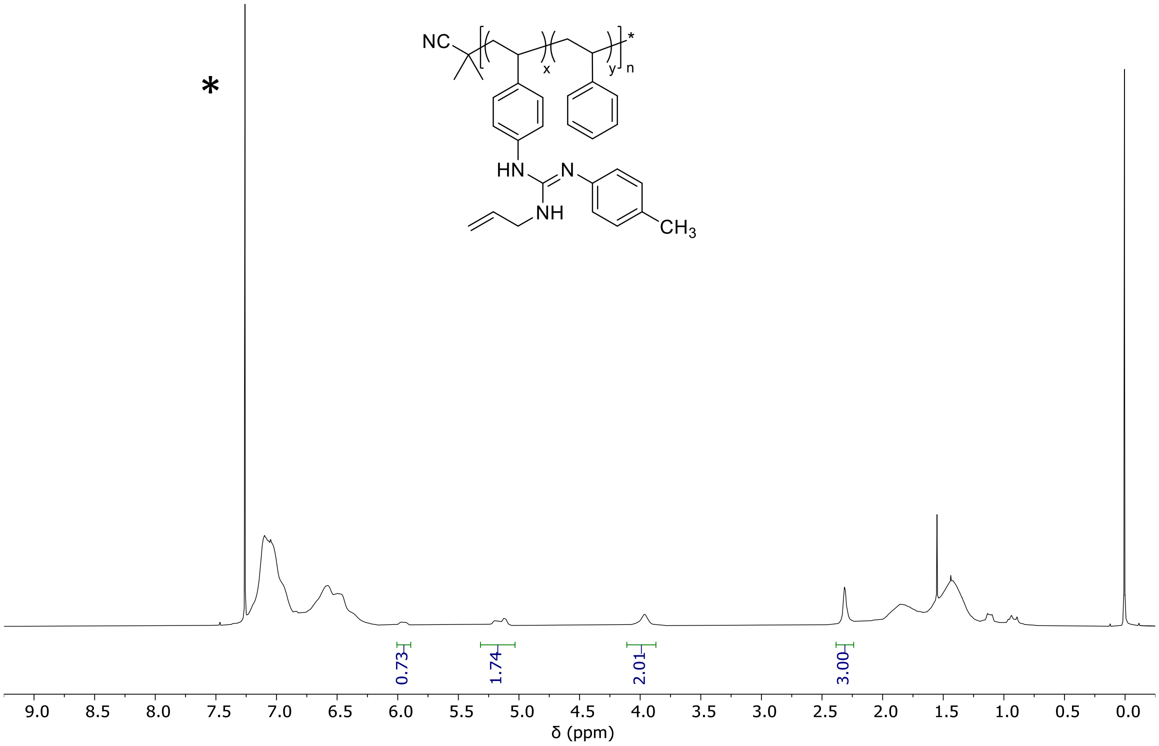

Figure S17. ^{1}H NMR spectrum (500 MHz, $CDCl_3$) and structure of poly(**1**-*co*-styrene)$_{10}$ modified with allyl amine. * = $CHCl_3$.

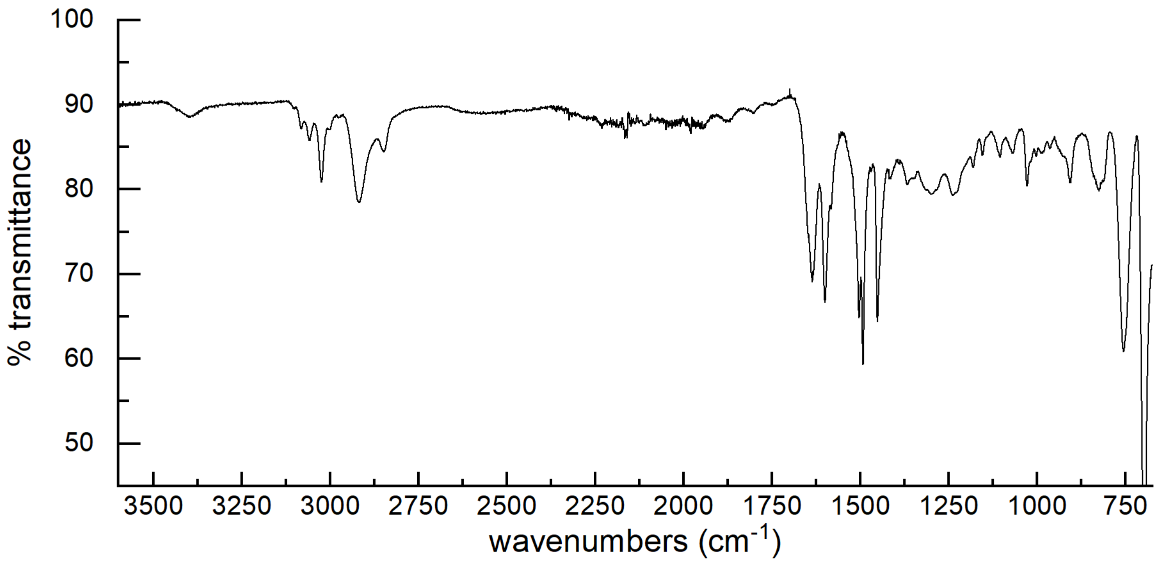

Figure S18. ATR FT-IR spectrum of poly(**1**-*co*-styrene)$_{10}$ modified with allyl amine.

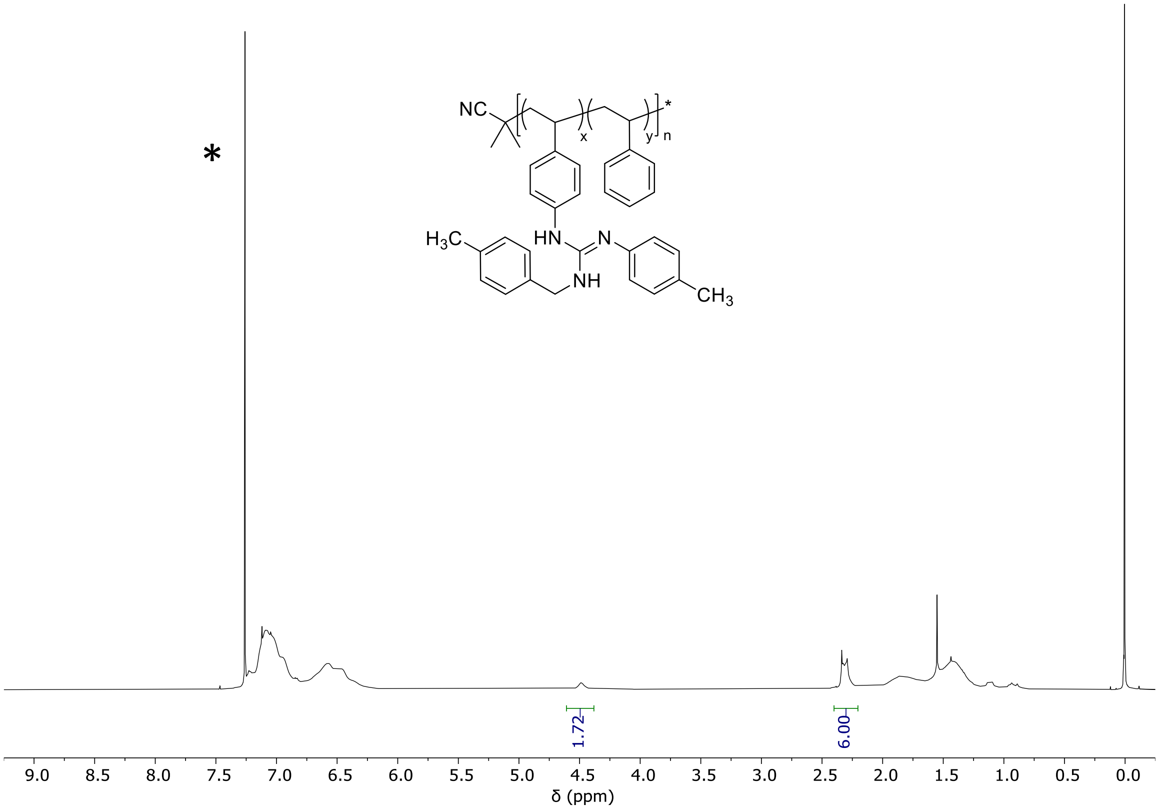

Figure S19. ^{1}H NMR spectrum (500 MHz, $CDCl_3$) and structure of poly(**1**-*co*-styrene)$_{10}$ modified with 4-methylbenzyl amine. * = $CHCl_3$.

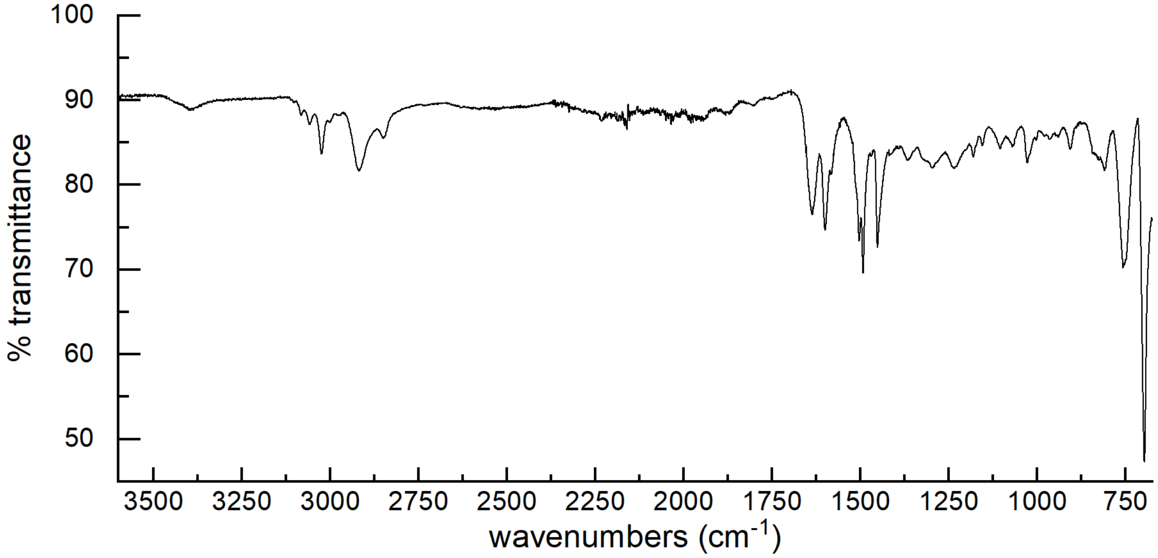

Figure S20. ATR FT-IR spectrum of poly(**1**-*co*-styrene)$_{10}$ modified with 4-methylbenzyl amine.

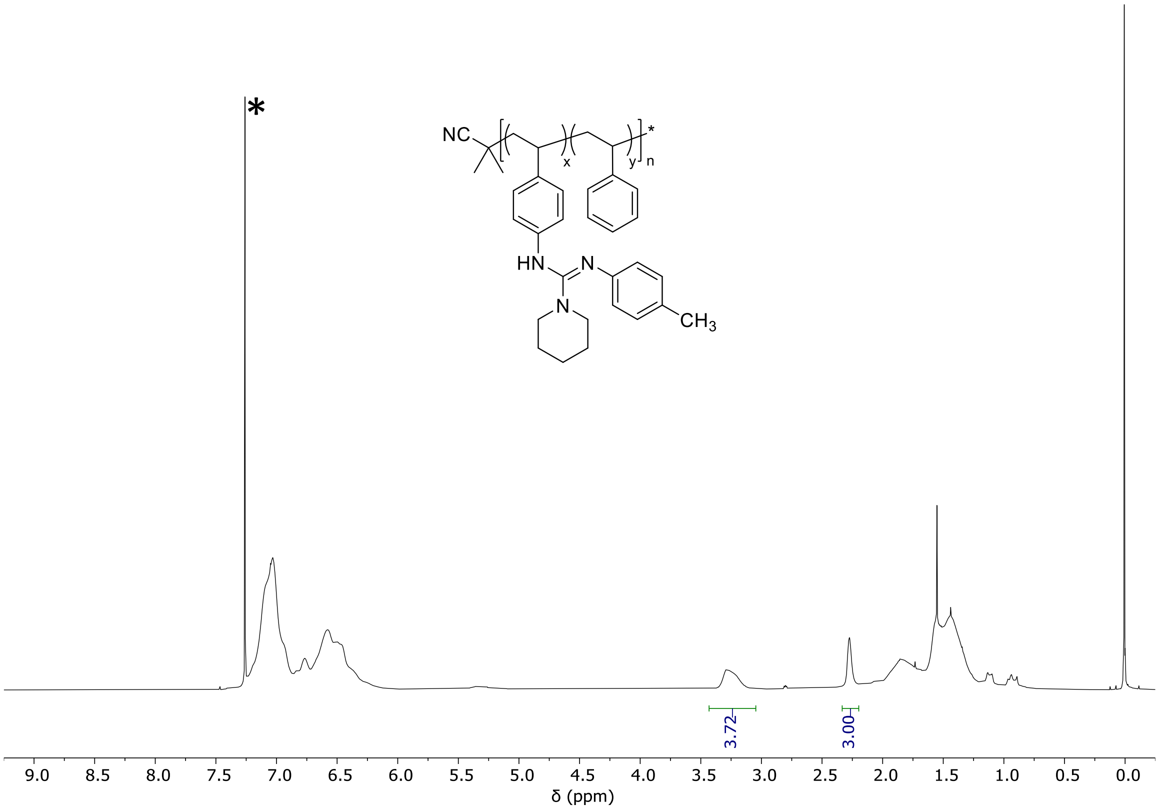

Figure S21. ^{1}H NMR spectrum (500 MHz, $CDCl_3$) and structure of poly(**1**-*co*-styrene)$_{10}$ modified with piperidine. * = $CHCl_3$.

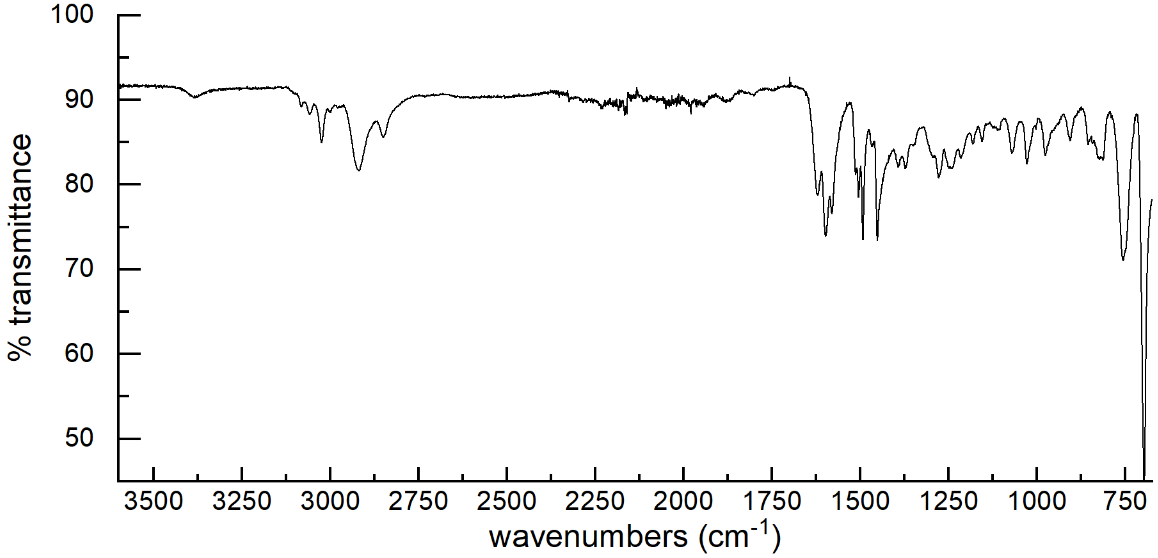

Figure S22. ATR FT-IR spectrum of poly(**1**-*co*-styrene)$_{10}$ modified with piperidine.

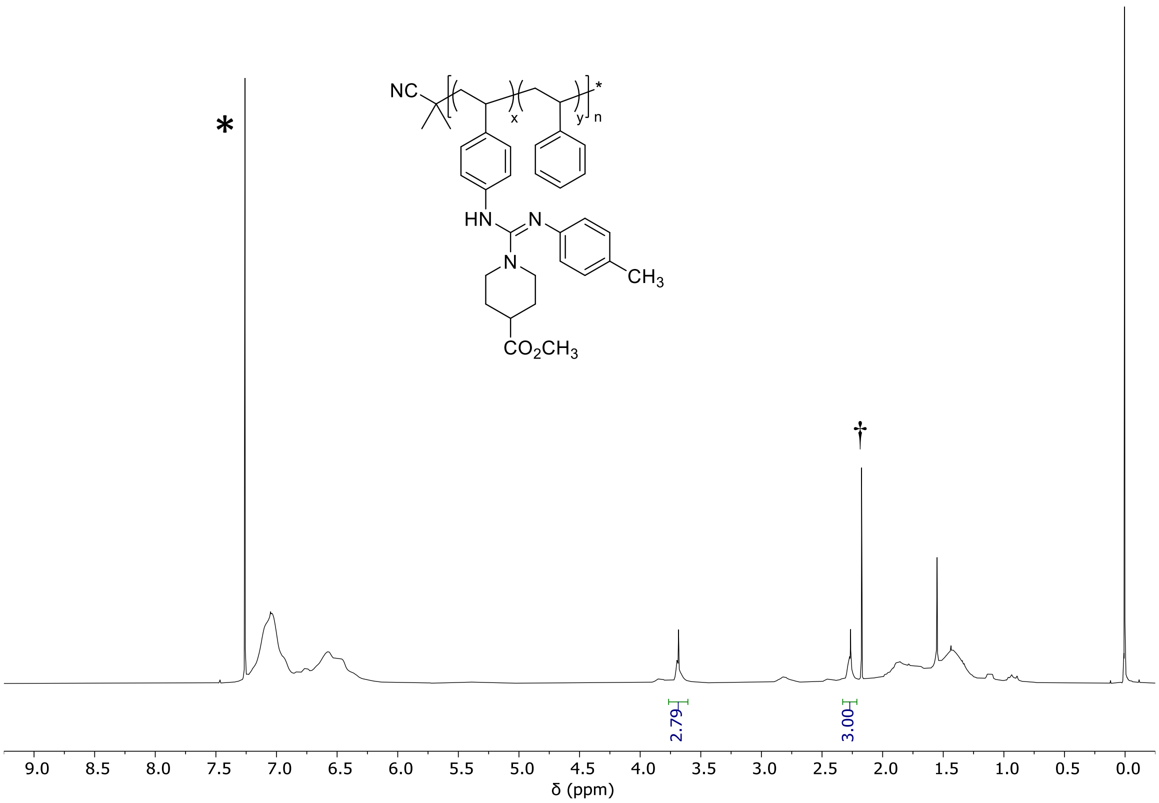

Figure S23. ^{1}H NMR spectrum (500 MHz, $CDCl_3$) and structure of poly(**1**-*co*-styrene)$_{10}$ modified with methyl piperidine-4-carboxylate. * = $CHCl_3$, † = acetone.

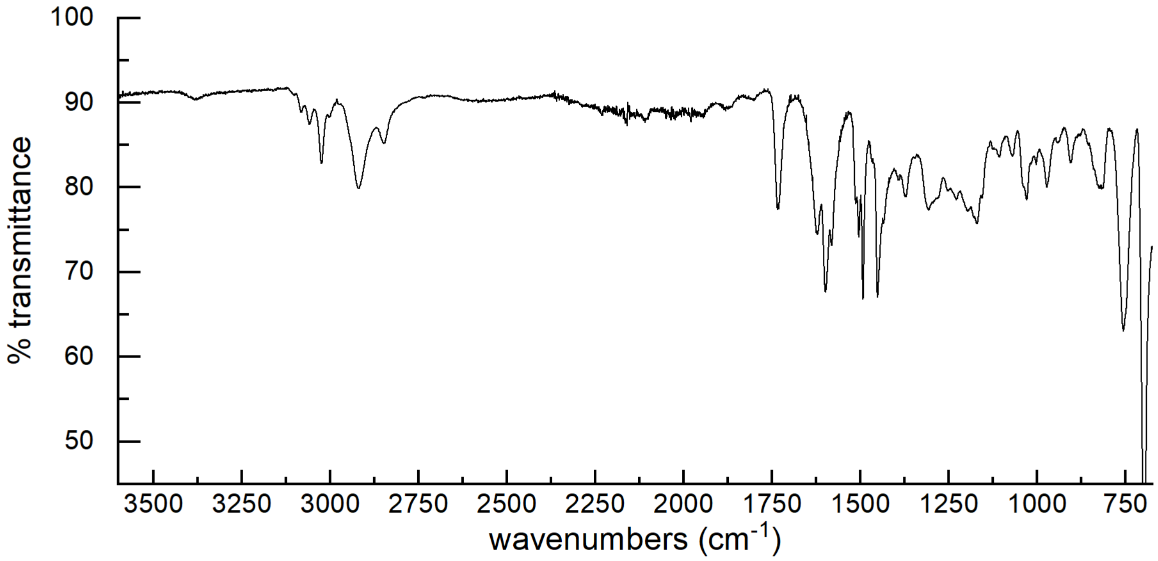

Figure S24. ATR FT-IR spectrum of poly(**1**-*co*-styrene)$_{10}$ modified with methyl piperidine-4-carboxylate.

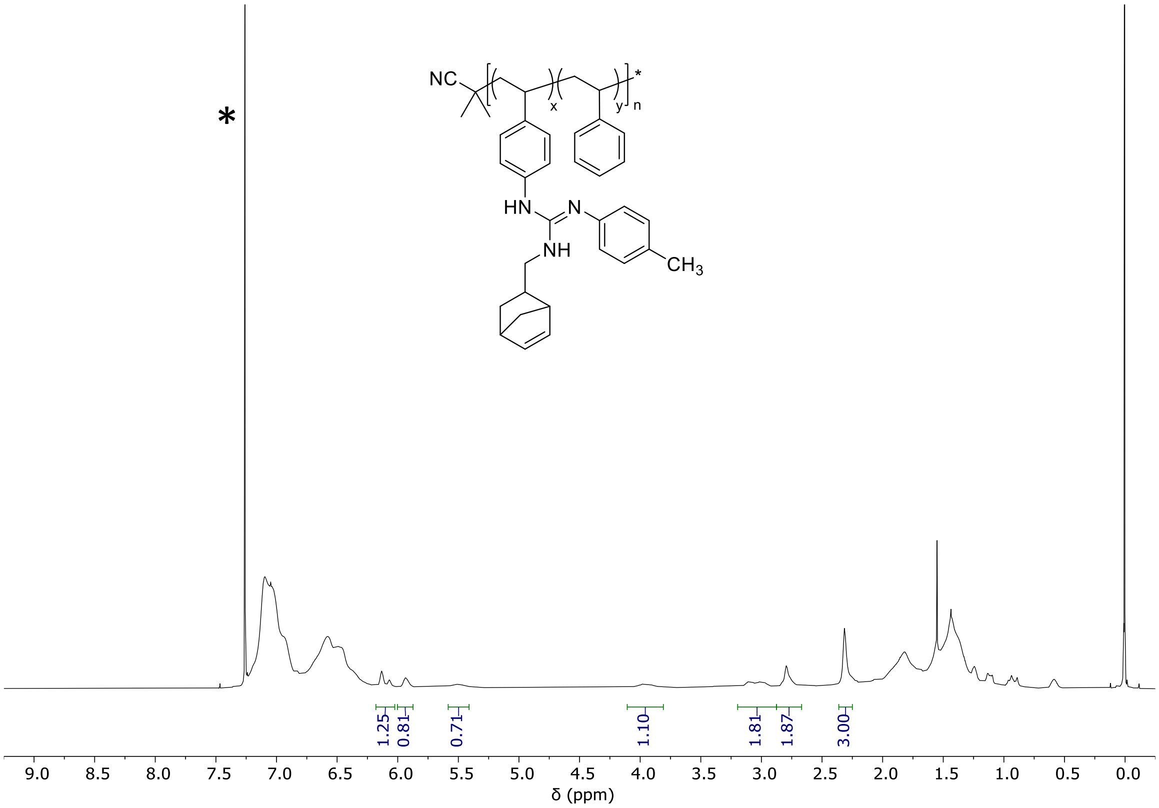

Figure S25. ^{1}H NMR spectrum (500 MHz, $CDCl_3$) and structure of poly(**1**-*co*-styrene)$_{10}$ modified with 5-norbornene-2-methylamine. * = $CHCl_3$.

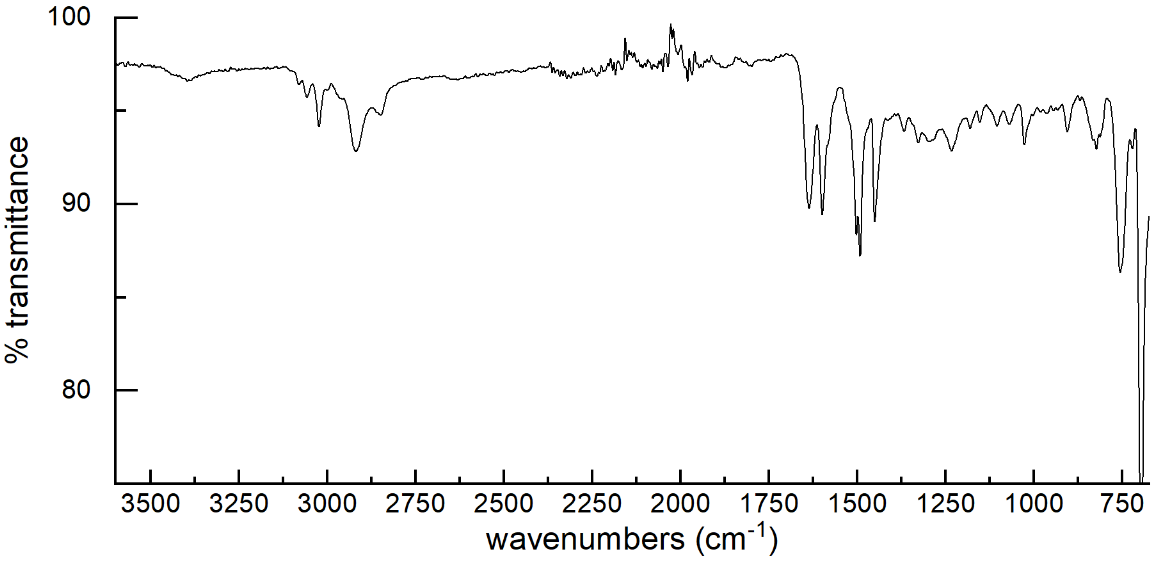

Figure S26. ATR FT-IR spectrum of poly(**1**-*co*-styrene)$_{10}$ modified with 5-norbornene-2-methylamine.

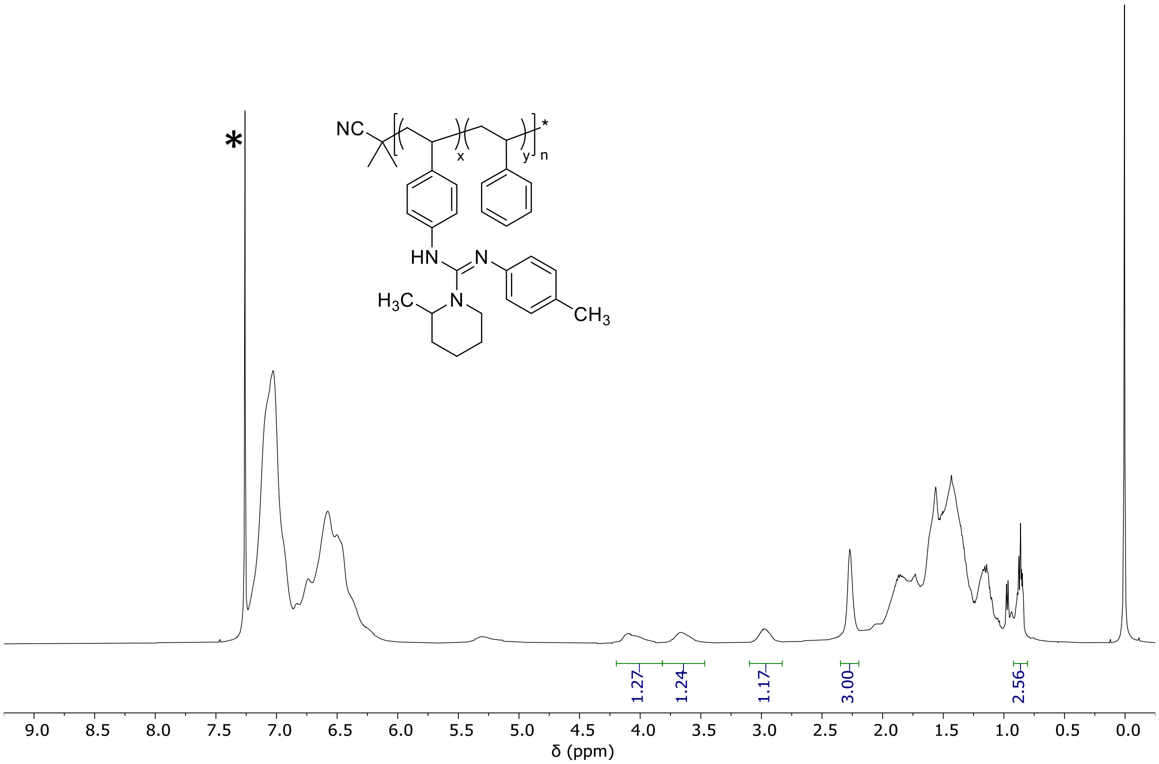

Figure S27. ^{1}H NMR spectrum (500 MHz, $CDCl_3$) and structure of poly(**1**-*co*-styrene)$_{10}$ modified with 2-methylpiperidine. * = $CHCl_3$.

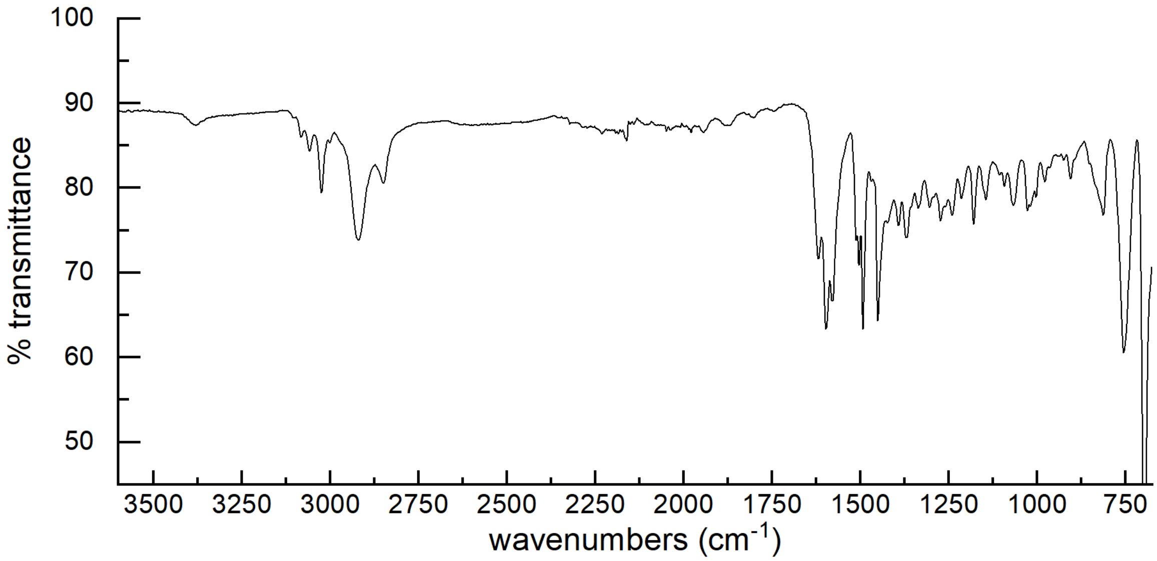

Figure S28. ATR FT-IR spectrum of poly(**1**-*co*-styrene)$_{10}$ modified with 2-methylpiperidine.

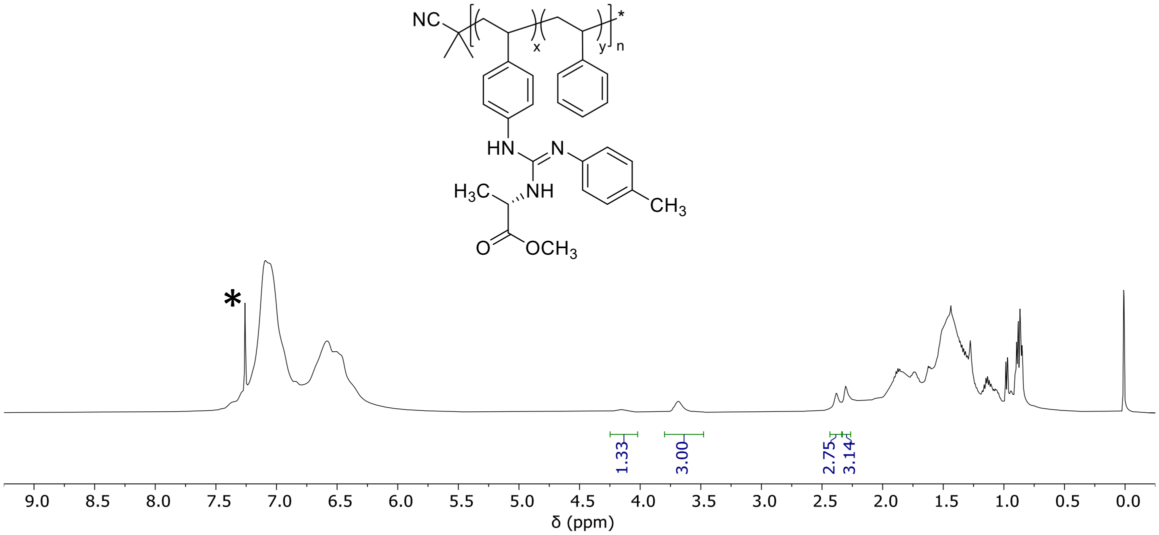

Figure S29. ^{1}H NMR spectrum (500 MHz, $CDCl_3$) and structure of poly(**1**-*co*-styrene)$_{10}$ modified with *L*-alanine methyl ester hydrochloride. * = $CHCl_3$.

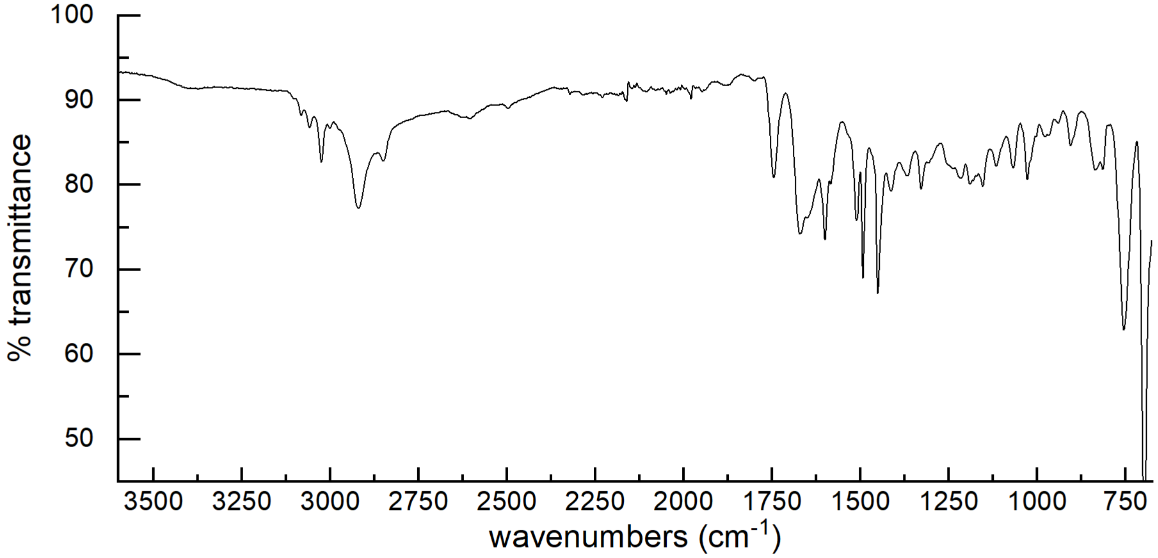

Figure S30. ATR FT-IR spectrum of poly(**1**-*co*-styrene)$_{10}$ modified with *L*-alanine methyl ester hydrochloride.

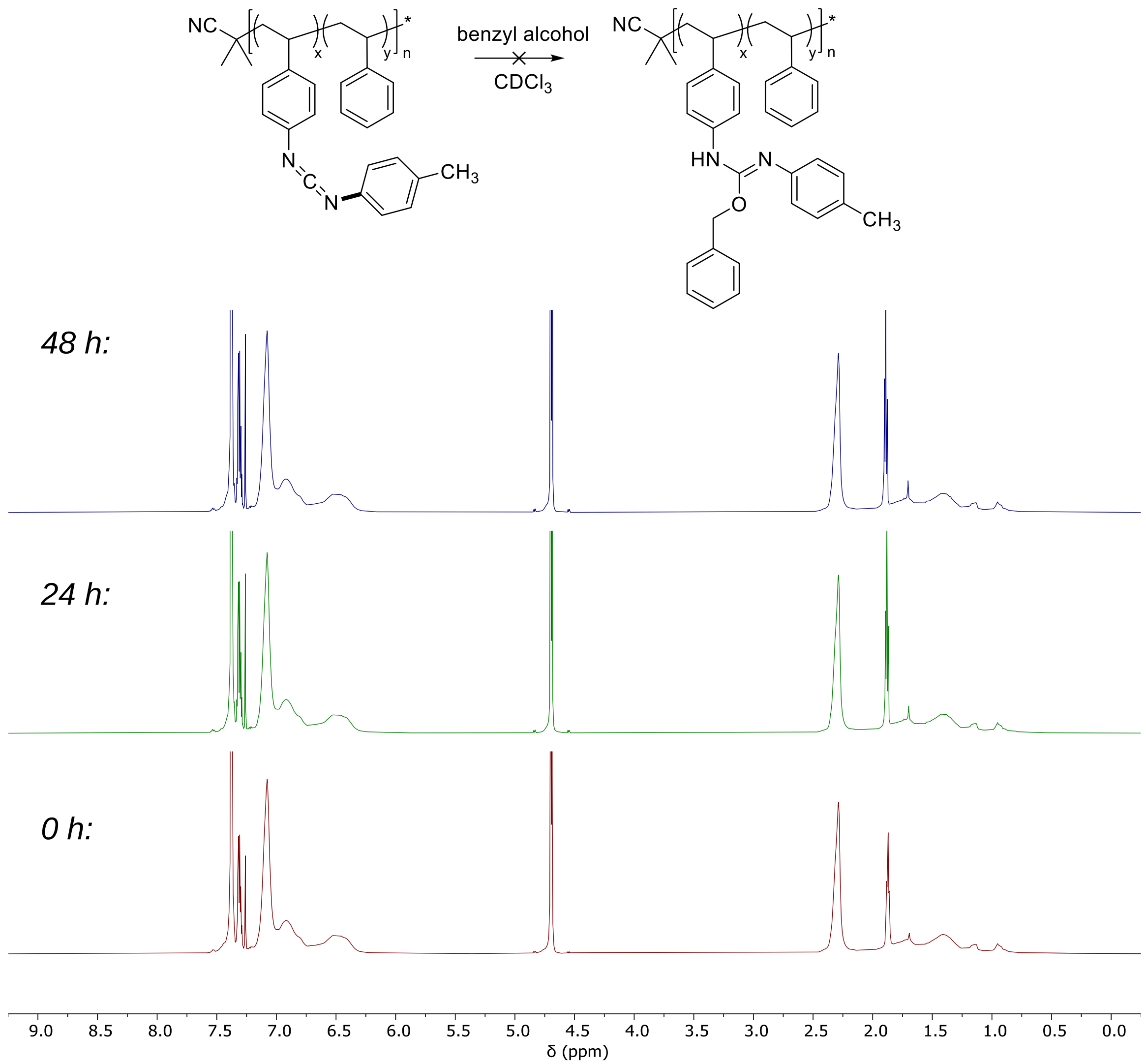

Figure S31. Examination of potential reaction of poly**1** with benzyl alcohol. ^{1}H NMR spectra (500 MHz, $CDCl_3$) of 1 equiv. benzyl alcohol (versus CDI repeat unit) combined with poly**1** indicate no reaction over the course of 48 h, as no significant changes are evident across the three spectra. Spectra are clipped vertically for clarity.

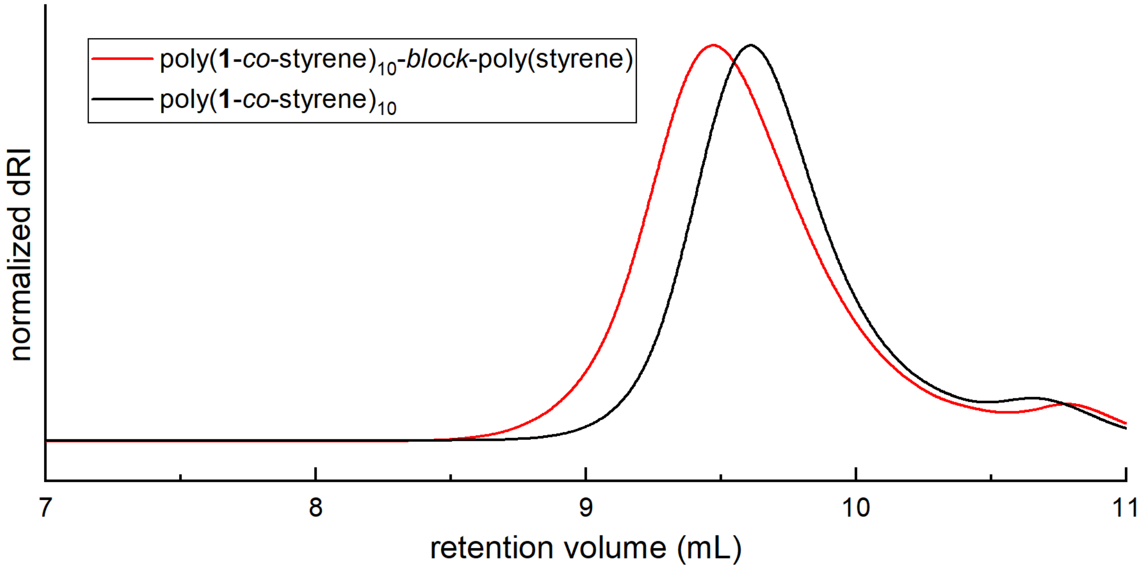

Figure S32. SEC chromatograms of poly(**1**-*co*-styrene)$_{10}$ obtained by RAFT copolymerization (black) and poly(**1**-*co*-styrene)$_{10}$-*block*-poly(styrene) obtained after reinitiation with additional styrene (red). Poly(**1**-*co*-styrene)$_{10}$ M_w 9 kDa, *Đ* 1.2; poly(**1**-*co*-styrene)$_{10}$-*block*-poly(styrene) M_w 36 kDa, *Đ* 1.2.

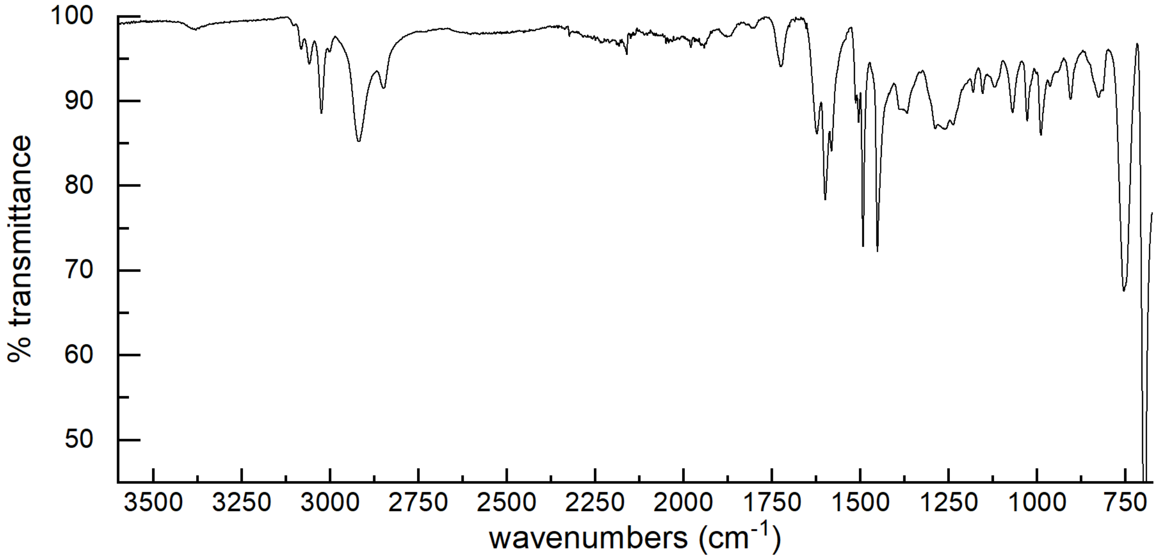

Figure S33. ATR FT-IR spectrum of CAN_5.

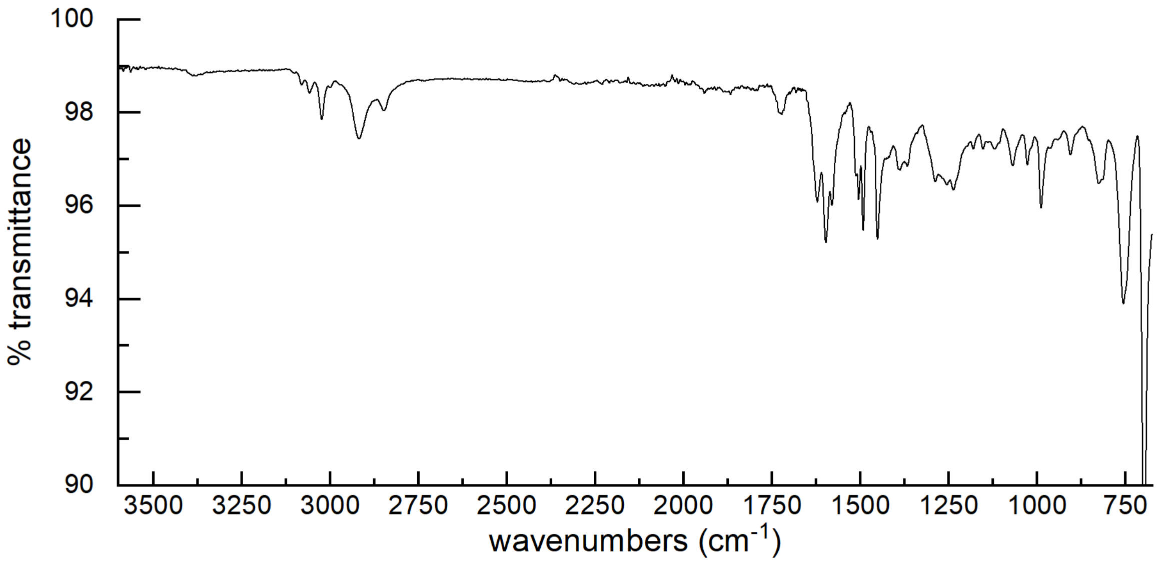

Figure S34. ATR FT-IR spectrum of CAN_{10}.

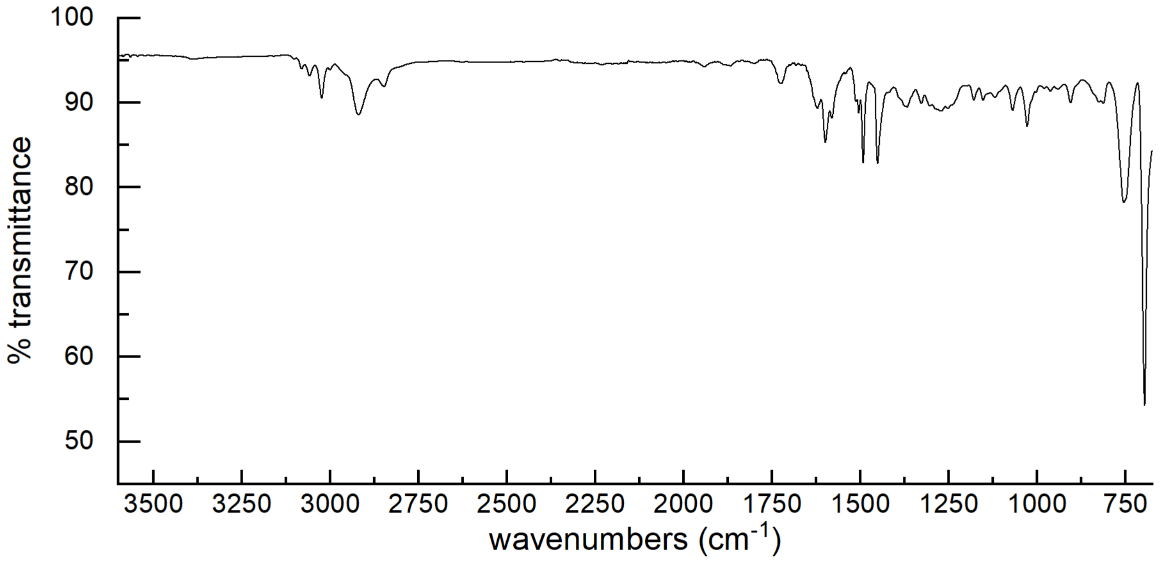

Figure S35. ATR FT-IR spectrum of dm-CAN_5.

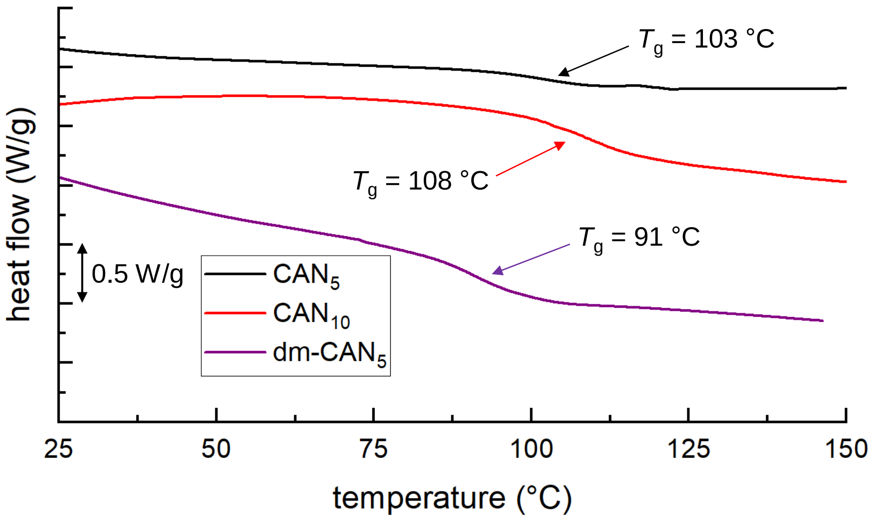

Figure S36. DSC thermograms (exo up) of CAN$_5$, CAN$_{10}$, and dm-CAN$_5$ (second heating, 15 °C/min, N_2 atmosphere). Data have been shifted vertically for clarity.

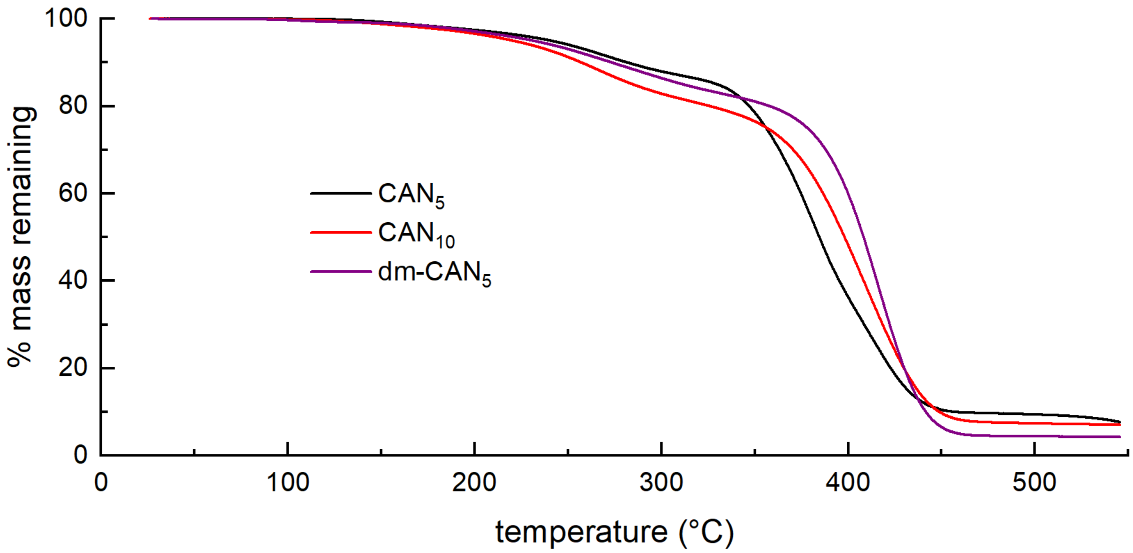

Figure S37. TGA thermograms of CAN$_5$, CAN$_{10}$, and dm-CAN$_5$ (20 °C/min, N_2 atmosphere). CAN$_5$ $T_{d,5\%}$ = 240 °C; CAN$_{10}$ $T_{d,5\%}$ = 220 °C; dm-CAN$_5$ $T_{d,5\%}$ = 231 °C.

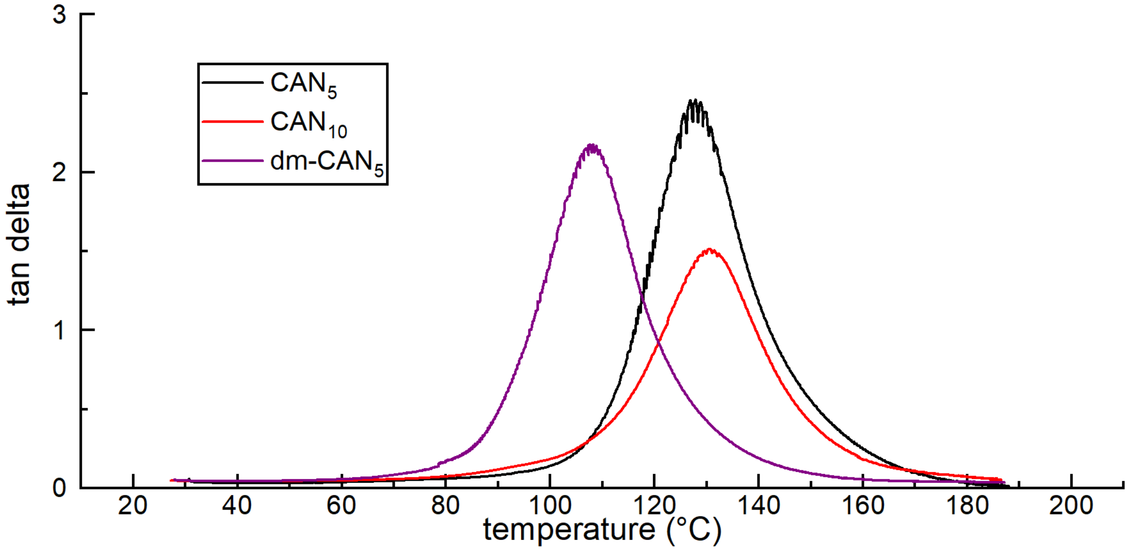

Figure S38. DMA thermograms of tan δ versus temperature for CAN$_5$, CAN$_{10}$, and dm-CAN$_5$. T_g is taken as the peak of tan δ (see Table S1 below for values).

Table S1. Compiled data from DMA temperature ramps (main text Figure 3b, Figure S38).

**Sample**	***E′*, 40 °C (MPa)**	***E′*, 170 °C (MPa)**	**T_g (°C)** [a]	**ν_e (mmol m^{-3})** [b]
CAN$_5$	1850	0.816	128	0.0765
CAN$_{10}$	3180	2.21	131	0.200
dm-CAN$_5$	2190	0.742	108	0.0671

[a]Peak of tan δ. [b]Calculated as *E′*/3RTd at 170 °C, assuming a density of 1 g cm^{-3}.

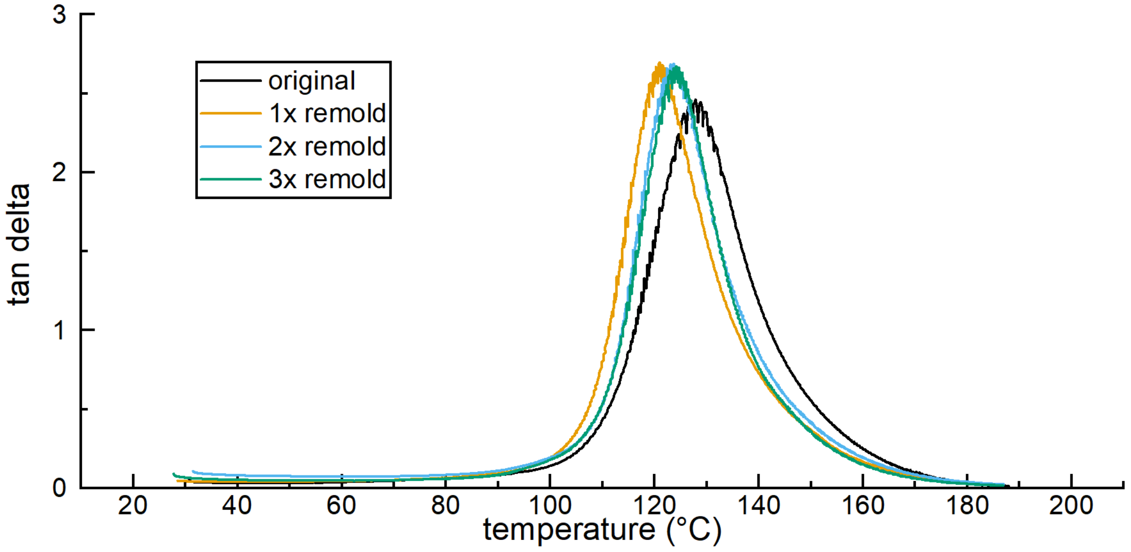

Figure S39. DMA thermograms of tan δ versus temperature and upon reprocessing the same sample up to three times. T_g is taken as the peak of tan δ (see Table S2 below for values).

Table S2. Compiled data from DMA temperature ramps of CAN_5 reprocessing experiments (main text Figure 3c, Figure S39).

Sample	*E′*, 40 °C (MPa)	*E′*, 170 °C (MPa)	T_g (°C) [a]	ν_e (mmol m^{-3})[b]
original CAN_5	1850	0.816	128	0.0765
first remold	1690	0.645	121	0.0584
second remold	1122	0.605	124	0.0547
third remold	1980	0.967	124	0.0875

[a]Peak of tan δ. [b]Calculated as *E′*/3RTd at 170 °C, assuming a density of 1 g cm^{-3}.

Table S3. Compiled data obtained from stress relaxation analyses of CAN_5, CAN_{10}, and dm-CAN_5.

Sample	Temperature (°C)	G_0 (MPa)[a]	τ* (sec)[b]	β[b]
#1 CAN_5	150	0.0246	9264	0.784
#1 CAN_5	155	0.0257	6072	0.826
#1 CAN_5	160	0.0261	4850	0.789
#1 CAN_5	165	0.0238	3402	0.829
#1 CAN_5	170	0.0186	2642	0.851
#1 CAN_5	175	0.0157	2088	0.791
#2 CAN_5	150	0.0344	9354	0.821
#2 CAN_5	155	0.0355	6778	0.828
#2 CAN_5	160	0.0363	4755	0.830
#2 CAN_5	165	0.0364	3325	0.856
#2 CAN_5	170	0.0355	2575	0.841
#2 CAN_5	175	0.0339	1957	0.820
#3 CAN_5	150	0.0386	8973	0.783
#3 CAN_5	155	0.0399	6846	0.763
#3 CAN_5	160	0.0406	4198	0.851
#3 CAN_5	165	0.0407	3195	0.835
#3 CAN_5	170	0.0399	2395	0.825
#3 CAN_5	175	0.0384	1795	0.819

[a] First measured relaxation modulus upon application of 1% strain. [b] Calculated by fitting each data set to a stretched exponential model: $G(t) = G_0\, e^{-(\frac{t}{\tau*})^{\beta}}$.

Table S3 continued.

Sample	Temperature (°C)	G_0 (MPa)[a]	τ* (sec)[b]	β[b]
#1 CAN_{10}	150	0.0375	4186	0.908
#1 CAN_{10}	155	0.0406	3272	0.861
#1 CAN_{10}	160	0.0424	2341	0.871
#1 CAN_{10}	165	0.0420	1721	0.856
#1 CAN_{10}	170	0.0397	1241	0.837
#1 CAN_{10}	175	0.0363	928	0.813
#2 CAN_{10}	150	0.0646	4231	0.827
#2 CAN_{10}	155	0.0731	2934	0.863
#2 CAN_{10}	160	0.0781	2172	0.855
#2 CAN_{10}	165	0.0792	1581	0.852
#2 CAN_{10}	170	0.0762	1148	0.836
#2 CAN_{10}	175	0.0685	825	0.822
#3 CAN_{10}	150	0.0352	4242	0.836
#3 CAN_{10}	155	0.0362	2583	0.868
#3 CAN_{10}	160	0.0351	2052	0.884
#3 CAN_{10}	165	0.0342	1484	0.854
#3 CAN_{10}	170	0.0316	1059	0.851
#3 CAN_{10}	175	0.0283	784	0.821

[a] First measured relaxation modulus upon application of 1% strain. [b] Calculated by fitting each data set to a stretched exponential model: $G(t) = G_0\, e^{-(\frac{t}{\tau*})^{\beta}}$.

Table S3 continued.

Sample	Temperature (°C)	G_0 (MPa)[a]	τ* (sec)[b]	β[b]
#1 dm-CAN_5	150	0.0592	7737	0.650
#1 dm-CAN_5	155	0.0554	4613	0.752
#1 dm-CAN_5	160	0.0507	3097	0.797
#1 dm-CAN_5	165	0.0465	2283	0.801
#1 dm-CAN_5	170	0.0425	1693	0.791
#1 dm-CAN_5	175	0.0397	1264	0.781
#2 dm-CAN_5	150	0.0688	7802	0.690
#2 dm-CAN_5	155	0.0614	4648	0.780
#2 dm-CAN_5	160	0.0543	3241	0.809
#2 dm-CAN_5	165	0.0484	2351	0.811
#2 dm-CAN_5	170	0.0432	1712	0.814
#2 dm-CAN_5	175	0.0395	1283	0.787
#3 dm-CAN_5	150	0.0614	5927	0.668
#3 dm-CAN_5	155	0.0573	3845	0.751
#3 dm-CAN_5	160	0.0512	2757	0.791
#3 dm-CAN_5	165	0.0471	2062	0.799
#3 dm-CAN_5	170	0.0431	1545	0.797
#3 dm-CAN_5	175	0.0407	1176	0.786

[a] First measured relaxation modulus upon application of 1% strain. [b] Calculated by fitting each data set to a stretched exponential model: $G(t) = G_0\, e^{-(\frac{t}{\tau *})^{\beta}}$.

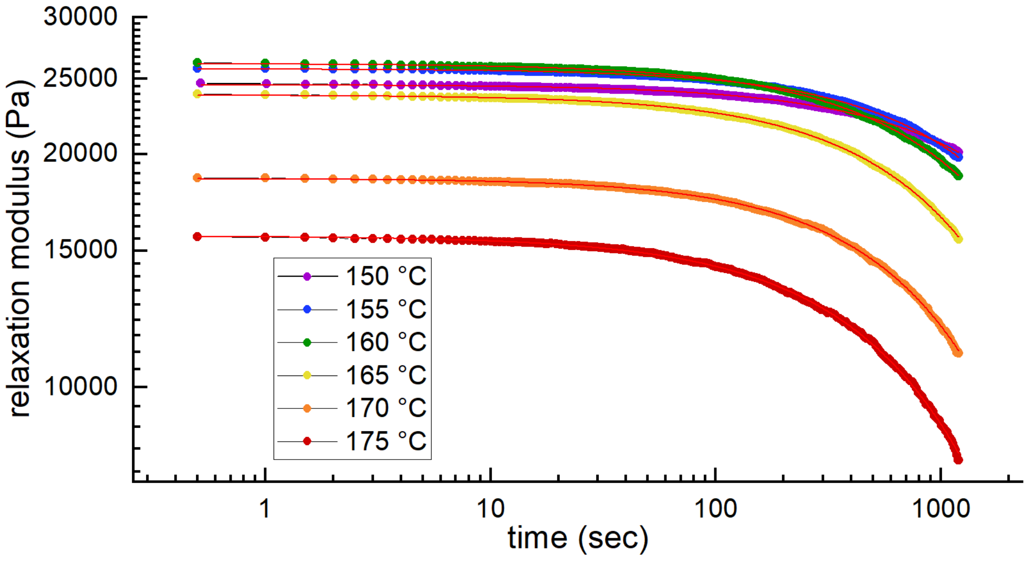

Figure S40. Non-normalized stress relaxation data for a representative sample of CAN_5. Fitting curves (red) calculated by fitting each data set to a stretched exponential model: $G(t) = G_0\ e^{-(\frac{t}{\tau*})^{\beta}}$.

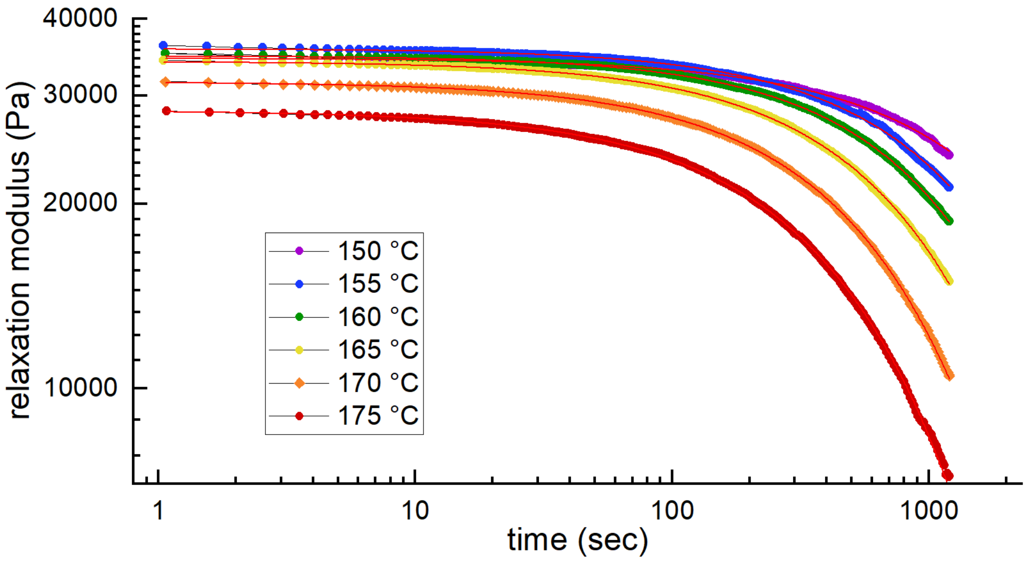

Figure S41. Non-normalized stress relaxation data for a representative sample of CAN_{10}. Fitting curves (red) calculated by fitting each data set to a stretched exponential model: $G(t) = G_0\ e^{-(\frac{t}{\tau*})^{\beta}}$.

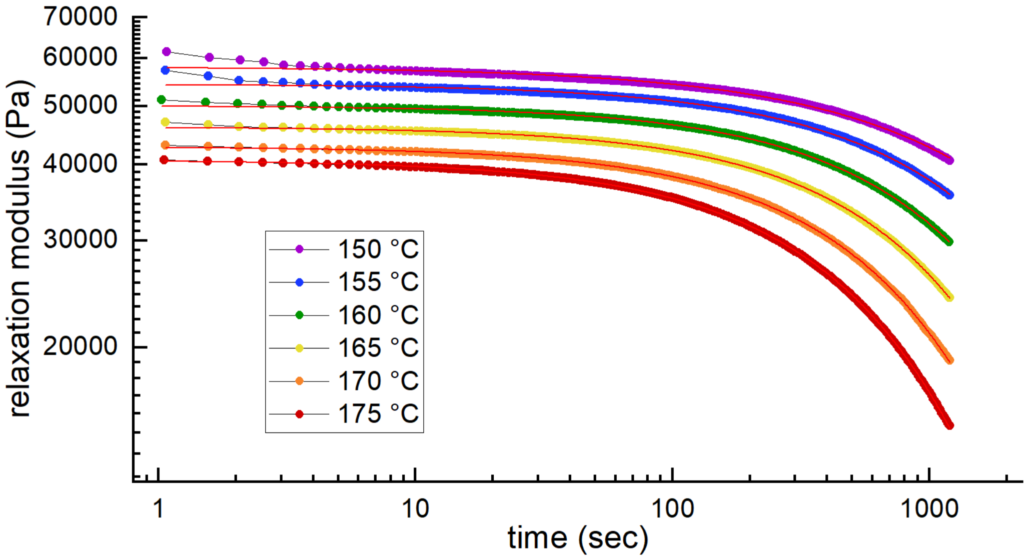

Figure S42. Non-normalized stress relaxation data for a representative sample of dm-CAN_5. Fitting curves (red) calculated by fitting each data set to a stretched exponential model: $G(t) = G_0\, e^{-(\frac{t}{\tau*})^{\beta}}$.

Table S4. Kinetics of TGM reaction in small molecule systems.

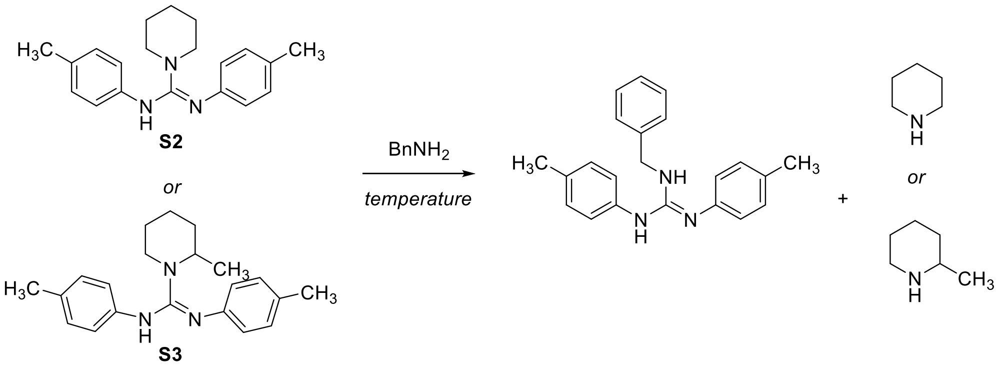

Entry	Guanidine	Temperature (°C)	k (s^{-1}) × 10^4
1	**S2**	130	4.83
2	**S2**	130	5.83
3	**S2**	130	6.30
4	**S2**	150	15.1
5	**S2**	150	17.4
6	**S2**	150	13.0
7	**S2**	160	34.0
8	**S2**	160	24.0
9	**S2**	160	27.0
10	**S3**	130	20.5
11	**S3**	130	21.0
12	**S3**	130	21.6
13	**S3**	150	48.2
14	**S3**	150	54.0
15	**S3**	150	55.1
16	**S3**	160	72.0
17	**S3**	160	62.0
18	**S3**	160	61.0

Initial reaction conditions: 1.5 – 1.6 M guanidine, 1.0 equiv. benzyl amine, 1.3 equiv. 1,3,5-trimethoxybenzene (internal standard).

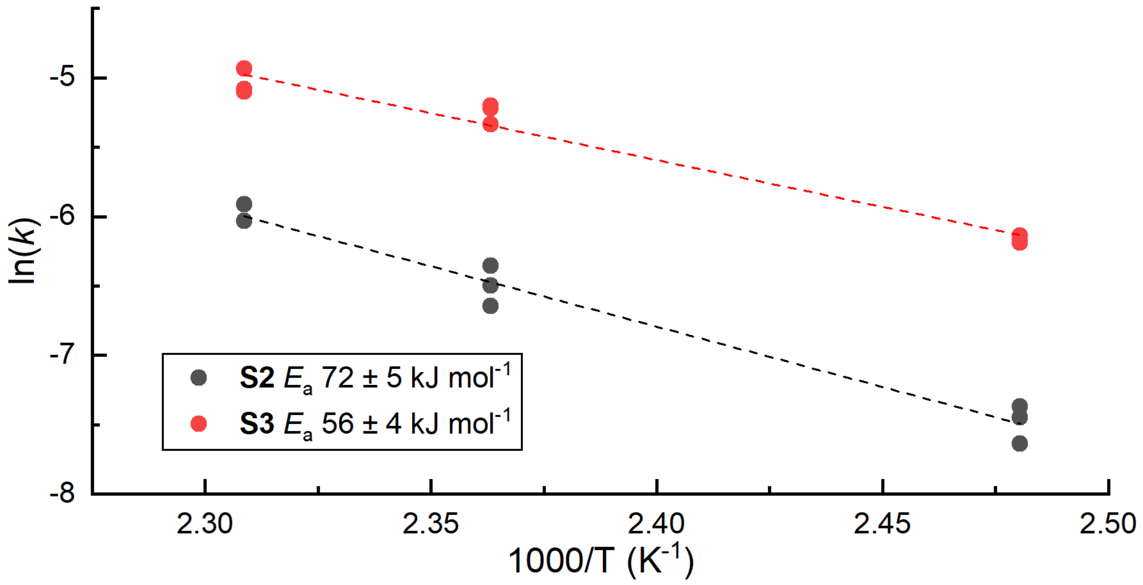

Figure S43. First-order Arrhenius plot of kinetic data for **S2** and **S3** presented in Table S4. Activation energies and standard errors are calculated from the linear lines of best fit (dashed) for each data set.

---

[1] Ramirez, V.; Van Pelt, E. B.; Pooni, R. K.; Melchor Bañales, A. J.; Larsen, M. B. Thermodynamic, kinetic, and mechanistic studies of the thermal guanidine metathesis reaction. *Org. Biomol. Chem.* **2022**, *20*, 5861-5868.
[2] Li, Y.; Hoskins, J. N.; Sreerama, S. G.; Grayson, M. A.; Grayson, S. M. The identification of synthetic homopolymer end groups and verification of their transformations using MALDI-TOF mass spectrometry. *J. Mass Spectrom.* **2010**, *45*, 587-611.

^{1}H and ^{13}C NMR spectra of compounds S1 and 1.

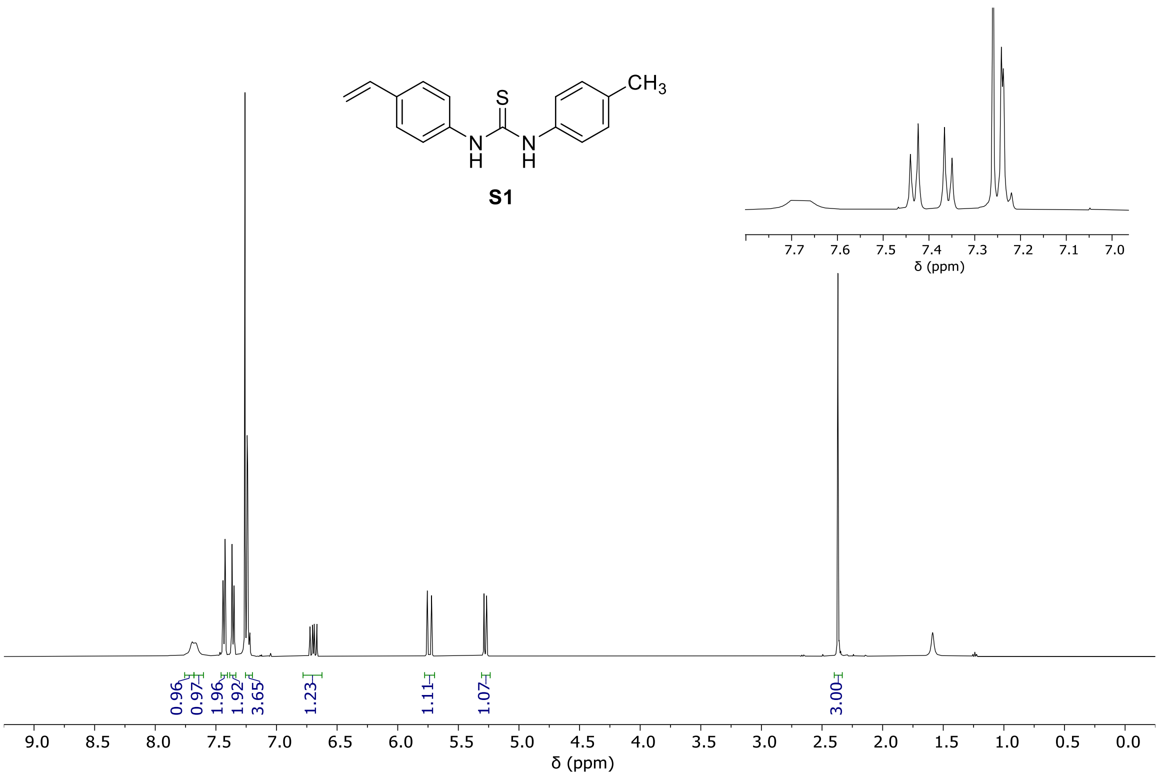

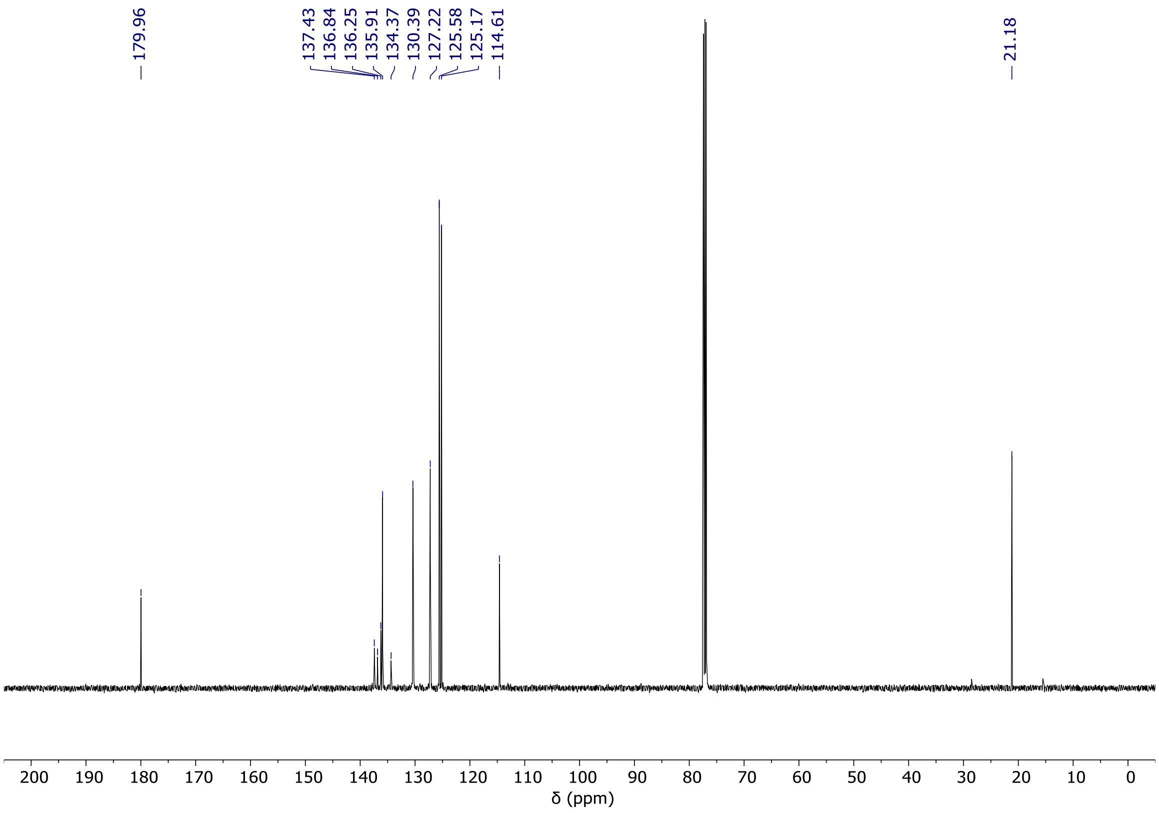

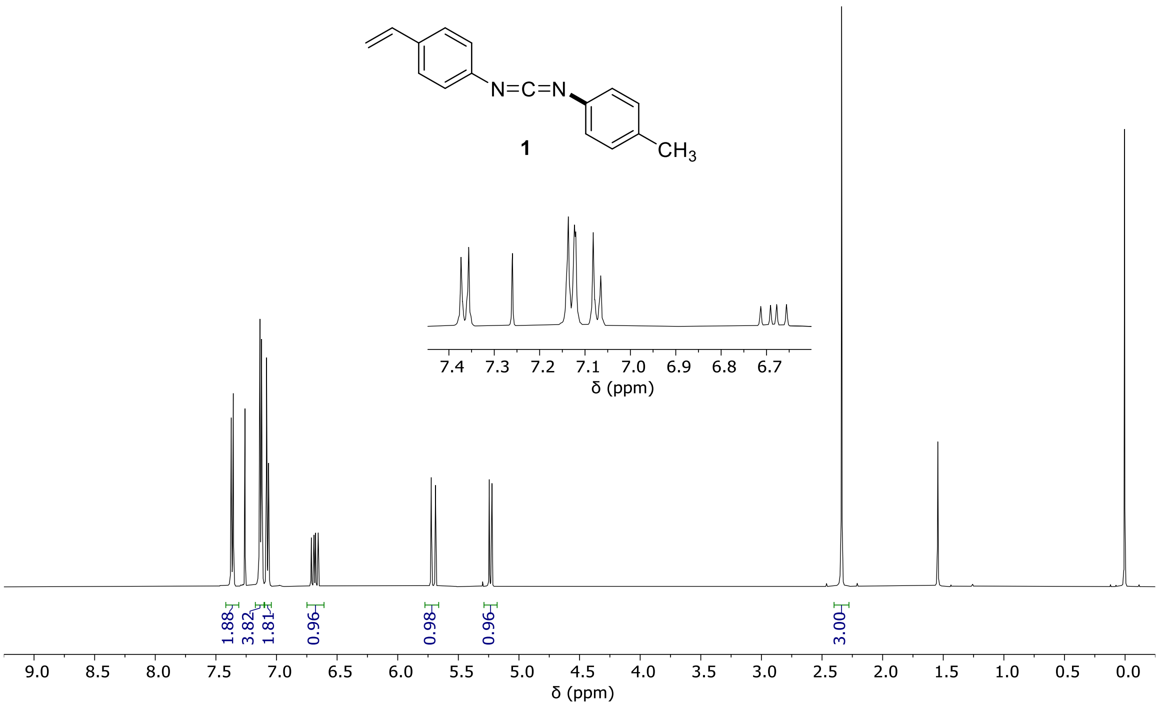
N=C=N
CH3
1
7.4 7.3 7.2 7.1 7.0 6.9 6.8 6.7
δ (ppm)
1.88
3.82
1.81
0.96
0.98
0.96
3.00
9.0 8.5 8.0 7.5 7.0 6.5 6.0 5.5 5.0 4.5 4.0 3.5 3.0 2.5 2.0 1.5 1.0 0.5 0.0
δ (ppm)

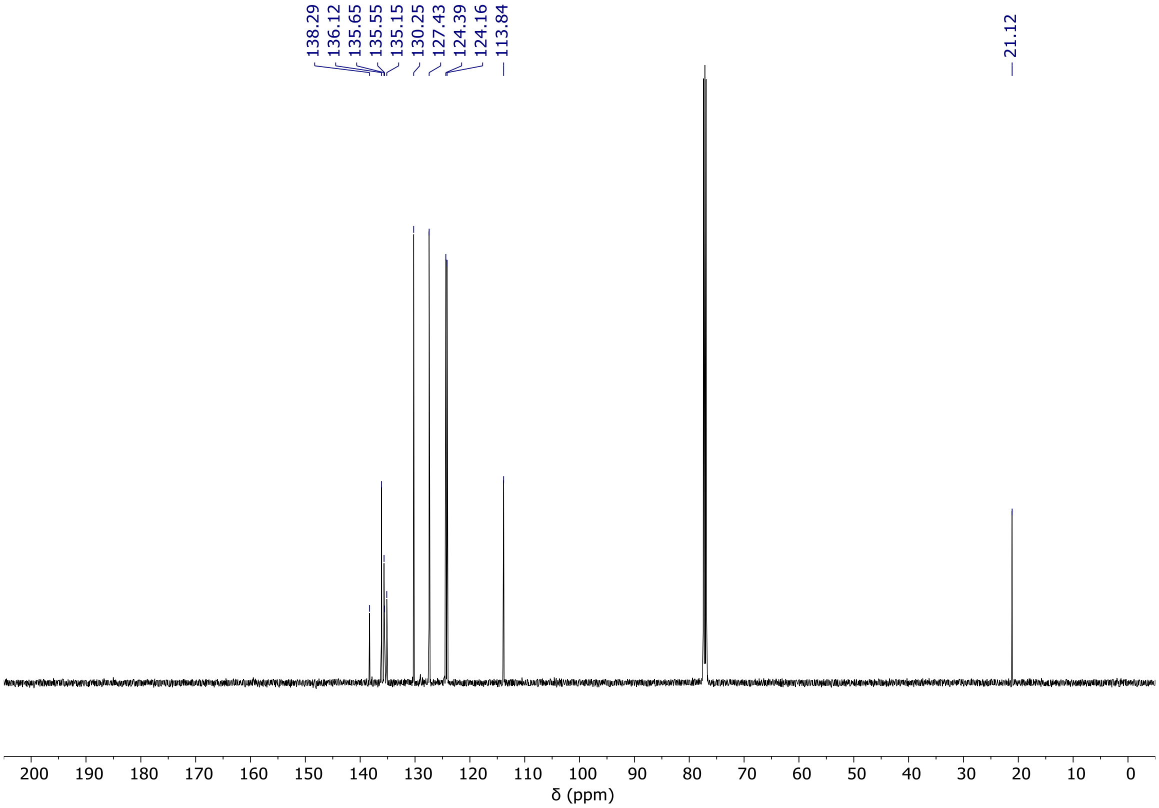
138.29
136.12
135.65
135.55
135.15
130.25
127.43
124.39
124.16
113.84
21.12
200 190 180 170 160 150 140 130 120 110 100 90 80 70 60 50 40 30 20 10 0
δ (ppm)
